# The interactions between dietary fats intake and *Caveolin 1 rs 3807992* polymorphism with fat distribution in overweight and obese women: a cross-sectional study

**DOI:** 10.1186/s12920-021-01114-7

**Published:** 2021-11-09

**Authors:** Yasaman Aali, Farideh Shiraseb, Faezeh Abaj, Fariba koohdani, Khadijeh Mirzaei

**Affiliations:** Department of Community Nutrition, School of Nutritional Sciences and Dietetics, Tehran University of Medical Sciences (TUMS), P.O. Box, 14155-6117 Tehran, Iran

**Keywords:** Obesity, Dietary fats, Fat distribution, Body composition, Caveola, *Caveolin 1*, Polymorphism, Interaction

## Abstract

**Background:**

It has been reported that dietary fats and genetic factors in individuals are associated with the pattern of fat distribution. This study aimed to evaluate the interaction between dietary fats intake and *Caveolin1 (CAV-1) rs 3807s992* polymorphism with fat distribution in overweight and obese women.

**Methods:**

A total of 221 participants were included in the current cross-sectional study. Body composition, biochemical parameters were evaluated by body composition analyzer and Pars Azmoon kits and genotypes determination was performed by PCR–RFLP, dietary fats were measured using a validated semi-quantitative food frequency questionnaire (FAQ).

**Results:**

The frequency of GG, AA and AG genotypes were 53.1, 24.6, and 22.3%, respectively, and the mean intake of total dietary fat intake was 97.47 ± 36.87 g. There was positive significant interaction between total fat intake and AA genotype on visceral fat level (*p* = 0.001), trunk fat (*p* = 0.01) and waist circumference (*p* = 0.05), positive significant interaction between total fat intake and AG genotype on the waist to hip ratio (WHR) (*p* = 0.02) and visceral fat level (*p* = 0.05), positive borderline significant interaction between saturated fatty acid and AA genotype on the trunk fat (*p* = 0.06), and between trans-fatty acids and AG genotype on WHR (*p* = 0.04), visceral fat level (*p* = 0.01), and between monounsaturated fatty acid and AG genotype on WHR (*p* = 0.04), and a borderline interaction between polyunsaturated fatty acid and AA genotypes on visceral fat level (*p* = 0.06), negative significant interaction between AG genotypes and linolenic acid on WHR (*p* = 0.04), borderline significant interaction between ALA and AG genotype on WHR (*p* = 0.06).

**Conclusions:**

Our findings showed that *CAV-1 rs 3807992* polymorphism and dietary fats were associated with fat distributions in individuals.

**Supplementary Information:**

The online version contains supplementary material available at 10.1186/s12920-021-01114-7.

## Introduction

Obesity is the biggest public health challenge of the current century and for this reason in many countries obesity and central obesity have become the main health issues [1, 2]. Prevalence of central obesity in developed countries was reported between 8.7% until 32.2% and in developing countries between 3.8% until 51.7%, and also the frequency of central obesity in Iranian women 34.4% [3, 4]. It is a multifactorial disease when the energy intake is more than the energy expenditure, excess energy store in fat tissue and causes obesity [5]. Various factors including genetic, metabolic, behavior, environmental, cultural factors, economic or social status affect the prevalence of obesity [6]. It has been reported that fat distribution in the body relative to body mass index (BMI) an important role in disorders metabolic including hypertension, diabetes type 2, cardiovascular disorders [7–9]. The ascending trend prevalence of central obesity in countries like Iran has been attributed to increased environmental risk factors including diet [10]. A low-fat, low-glycemic index diet increases satiety, reduces insulin secretion, improves insulin sensitivity, and increases weight control, thereby affecting the distribution of fat in the body [11, 12]. One of the most important diet factor effects on fat distribution is the dietary fat intake for instance omega 3 polyunsaturated fatty acids (PUFA), that with inhibition lipogenic enzyme and rise beta-oxidation cause reduce fat content in fat tissue [13]. Omega 3 PUFA from marine sources more than its plant sources like alpha-linolenic acid (ALA) cause decrease fat in tissue [14]. Maybe the consumption of various oils has associated with the fat distribution in the body, such olive oil, which is a rich source of oleic acid, and in individuals that consume olive oil, the prevalence of obesity is low than to individuals that consume sunflower oil [15]. Other dietary fat like linoleic acid may improve insulin sensitivity by reducing fat accumulation in muscle and pancreas [16, 17]. Excessive consumption of PUFA is related to waist to hip ratio (WHR) [7, 18]. Despite the diet, obesity may arise from the interactions of multiple genes [19]. Apart from diet some of the genotype polymorphism have a key role in fat distribution meta-analysis studies about genome-wide association studies (GWAS) reported that 97 and 49 genetic loci were related to BMI and WHR [20, 21]. One of the genes that could possibly affect a person's body weight is *Caveolin1 (CAV-1)*, so that its expression in the adipose tissue is augmented in obese patients with or without type 2 diabetes mellitus that this could be due to the increased transport of fatty acids to the plasma membrane [22]. Caveolins are a family of integral membrane proteins that are the main membrane components of Caveolae, and associated with various human diseases such as breast cancer, brain tumors, inflammation, and obesity [23–25], and *CAV1* is a 22 kDa protein and located on chromosome 7q31.2, and also encoded by a 36.4 kb gene [26]. Has been reported that single nucleotide polymorphism (SNP) rs3807992 is located at the intronic region of the CAV1 gene and may alter the expression and function of the CAV-1 gene through mRNA regulation [27]. Studies have shown that *CAV-1 rs 926198* is associated with MetS in Caucasians and the Hispanic population and the *rs 3807989* SNP with the risk of coronary heart disease in the Chinese Han population [28–30]. Changes in *CAV-1 rs11773845* and *rs 926198* are related to high serum triglyceride (TG) levels, metabolic syndrome and WHR [31]. It has been reported that in the human population, genetic variations in *CAV-1* have been related to obesity and metabolic disorder, and also depletion of *CAV-1* and reduction in the number of caveolae have been related to diseases including cancer and cardiovascular and pulmonary diseases [32–35].

There have been many studies on the relationship between diet patterns and types of obesity, but there are a few studies that have been done on the relationship between dietary intake especially fat intake and body fat distribution pattern, and also *CAV-1* polymorphism. This study aimed to survey the the interactions between dietary fats intake and rs3807992 of the CAV-1 gene with fat distribution in overweight and obese women.

## Materials and methods

### Study population

This cross-sectional study was performed on 221 obese and overweight women 18–48 years old reared to the health-care centers were enrolled in the study by multistage random sampling, according to inclusion and exclusion criteria. Participants were enrolled in the study according to inclusion criteria included consent to participate in the study, female gender, being healthy overweight and obese with body mass index (BMI) between ≥ 25 and ≤ 40 (overweight: 25–29, obesity: 30–40), exclusion criteria included reluctance to cooperate in the study, pregnancy, lactation, and menopause, use of lipid and blood sugar lowering or weight loss pills, alcohol consumption, smoking, history of high blood pressure, diabetes mellitus, renal, liver, cardiovascular and cancer disease, adherence to weight loss diets and following an arbitrary special dietary regimen (such as diabetes, kidney and cardiovascular diets), weight oscillation in recent months.

Participants whose reported daily energy intakes higher than 4200 kcal/day or lower than 800 kcal/day were also excluded [36]. The protocol of the study was approved by the ethics committee of Tehran University of Medical Sciences (Ethics number: IR.TUMS.VCR.REC.1398.142) and all methods were carried out in accordance with relevant guidelines. All participants of study completed a written informed consent.

### Dietary intake assessment

We used an FFQ for assessing the usual dietary intakes of the past year of participants. The FFQ was a semi-quantitative questionnaire with 147 food items listed that had been validated by previous [37]. This questionnaire was completed by a trained dietitian. Participants reported their frequency of consumption of a given serving of each food item during the previous year on a daily, weekly, monthly, or yearly basis. Portion sizes of the consumed foods were converted to grams using household measurements [38] and then individuals' dietary intake data were analyzed using the Nutrition IV software.

### Body composition

We used a body composition analyzer (model BC-418 MA; Tanita, UK) to assess the body composition of all the participants. Body composition components, including body fat mass (FM), body fat percentage, visceral fat mass, truncal fat mass, legs, and arms fat mass, BMI, and fat mass index (FMI) by assay using bioelectric impedance analysis(BIA) [39].

### Anthropometric measures

Anthropometric parameters such as height, WC, and hip circumference (HC) were measured for participants by trained dietitians. Weight was measured using BIA, and also height with an accuracy of 0.5 cm by a Seca scale without shoes with shoulders in a normal standing position, in contact with the wall with their head, shoulders, heels, and hips, and their height. WC was measured in the narrowest area of the waist while individuals were at the end of a normal exhalation by a non-elastic tape with the accuracy of 0.5 cm and neck circumference (NC) was measured by tape with the accuracy of 1 mm. HC in the most prominent part was measured with an accuracy of 0.5 cm. To measure the arm circumference in a contracted position, keep the arm in line with the body and bend the elbow 90° upwards and wrap the meter around its most prominent part by caliper. WHR calculated as WC (cm) divided by HC (cm).WHtR is calculated as WC (cm) divided by height (cm).

### Assessment of physical activity and other covariates

Individuals’ physical activity was appraised using a reliable and validated International Physical Activity Questionnaire (IPAQ) [40]. It includes 7 questions that showed physical activity rate (vigorous, moderate, walking, and inactive), and also this questionnaire completed through interview. Each question consisted of two sectors the frequency of repetition of each movement per week and duration. From multiplying these two numbers for each of the levels of severe, moderate, and walking are obtained numerically, which according to the valid instructions, we multiply the number as a coefficient for the previously obtained number in three levels of severe, moderate, and walking. In the end, specific numbers in each level are added for each individual, which is the equivalent index of metabolic activity (Metabolic Equivalents: MET). Medical history and current use of medications and supplement history, smoking habits were obtained with questionnaires. Information about demographic characteristics (age, education, occupation, and marital status) was completed by the researcher using a demographic questionnaire.

### Blood sampling

Participants in this study were referred to the Nutrition and Biochemistry Laboratory of the school of Nutritional and Dietetics at Tehran University of medical sciences laboratory. 12 cc of venous blood samples were taken who fasted for 10–12 h. Blood samples were collected in two tubes (one tube contained EDTA anticoagulant and the other tube lacked this substance). Centrifuged for 15 min at 3000 rpm, and the remaining blood was washed three times with 0.9% NaCl solution. After serum separation, it was kept at − 80 °C for laboratory assessments.

### Laboratory measurements and HOMA-IR assessment

Fasting blood sugar was assayed by glucose oxidase phenol 4-amino antipyrine peroxidase (GOD-PAP) method. Serum TG level was measured with triacylglycerol kits by using glycerol-3-phosphate oxidase phenol 4-amino antipyrine peroxidase (GPO-PAP) method. Total cholesterol (CHOL) levels were assayed by the enzymatic endpoint method. Low-density lipoprotein-cholesterol (LDL-C) and high-density lipoprotein-cholesterol (HDL-C) were assayed by direct enzymatic clearance. All evaluations were performed using Pars Azmoon laboratory kits (test Pars Inc, Tehran, Iran). Insulin resistance (mIU/ml) was calculated by the homeostatic model assessment (HOMA). HOMA-insulin resistance calculated according to the following equation: [fasting plasma glucose (mmol/l) × fasting plasma insulin (µIU/l)]/22.5 [41].

### Genetic examination

DNA extraction is a sensitive step in determining genotype. In this study DNA extraction from blood samples by DNA extraction kit with Brand Mini Columns, Type G This DNA molecule was investigated as a pattern for amplification of single nucleotide polymorphism coding sequences. The concentration of extracted DNA was measured using the NanoDrop (Thermo Scientific Company, USA). A Survey of *rs3807992 CAV-1* polymorphism was conducted by PCR–RFLP. To ensure PCR performance, electrophoresis of PCR products was performed on the agarose gel. Importantly, 10% of the samples were directly sequenced for confirmation of the PCR–RFLP results. The sequencing process performed using the ABI PRISM 3730 automated sequencer (Applied Biosystems, Foster City, Calif, USA) [42]. The sequence of primers used is as follows: primers forward: 5-AGTATTGACCTGATTTGCCATG-3; reverse: 5-GTCTTCTGGAAAAAGCACATA-3. For enzymatic digestion of PCR *CAV-1*, we need to add 0.5 µl of Hin 1 II (NIaIII) enzyme, 2 µl of G buffer, 7 µl of PCR product, and 5 µl of mineral oil (at 37 °C for overnight) and placed the obtained product in Bain MarieAs a result to stop the enzymatic action, after removing the product from the Bain Marie, it must reach a temperature of 65° for 20 min. Pieces containing 3 genotypes were distinguished: GG, AA, and AG (Additional file 1).

### Statistical analyses

All statistical analysis was performed using the IBM SPSS software version 25.0 (SPSS, Chicago, IL, USA) and a *p* value < 0.05 was considered statistically significant. The Kolmogorov–Smirnov test was used to determine the normal distribution of the variables. A one-way analysis of variance (ANOVA) test was used to analyses variables among genotypes and types of dietary fats. Chi-square test was used to compare qualitative variables between groups. Also analysis of covariance (ANCOVA) was used to adjust the confounding variables (age, physical activity, energy intake, and BMI). For the survey, the interaction of genotypes and diet fats in quantitative variables from generalized linear models (GLMs) were used. Results were presented as Beta (B) and 95% confidence intervals (CIs). And the reference group was GG genotype. Bonferroni post hoc analysis was obtained for detecting significant mean difference of variables among tertiles.

## Result

### Study population characteristics

Our study was conducted on 221 obese and overweight Iranian women 18–48 years old. The overall prevalence of rs3807992 genotypes in participants for AA, AG, and GG was 24.6%, 22.3%, and 53.1%, respectively.The mean age, weight, BMI, and intake of total dietary fat intake were 35.58 ± 9.57 years, 79.62 ± 11.24 kg, 30.76 ± 3.92 kg/m^2^, and 97.47 ± 36.87 g, respectively (Table 1).

**Table 1:** Baseline characteristics of participants

Variables	Mean	SD
Age (year)	35.58	9.57
Weight (kg)	79.62	11.24
BMI (kg/m^2^)	30.76	3.92
FM (kg)	34.45	8.78
FMI (kg/m)	13.29	3.31
WC (cm)	98.50	9.69
WHR (cm)	1.34	6.12
WHtR (cm)	0.61	0.05
*Intake of dietary fat types*		
Intake of total dietary fat (g)	97.47	36.87
Intake of SFA (g)	28.89	11.67
Intake of TFA (g)	0.00	0.001
Intake of PUFA (g)	20.89	10.30
Intake of MUFA (g)	33.32	13.59
Intake of linoleic acid (g)	18.29	9.73
Intake of ALA (g)	1.20	0.69
Intake of EPA-DHA (g)	0.11	0.14

All data are presented as mean and SD

*BMI* body mass index, *FM* fat mass, *FMI* fat mass index, *WC* waist circumference, *WHR* weight to hip ratio, *WHtR* weight to height ratio, *SFA* saturated fatty acid, *TFA* trans-fatty acid, *PUFA* polyunsaturated fatty acid, *MUFA* monounsaturated fatty acid, *ALA* alpha-linolenic acid, *EPA-DHA* eicosapentaenoic acid and docosahexaenoic acid

### Association between characteristics of study population across genotypes of *CAV-1*

Table 1 showed an association between participant characteristics and CAV-1 genotypes. Participants were divided into 3 groups GG (n = 117), AG (n = 51) and AA (n = 53) based on *CAV-1 rs 3807992* genotypes. There was significant mean difference for age (*p* = 0.03) and economic status (*p* = 0.03) among genotypes and borderline significant difference in TG levels (*p* = 0.06), in the crude model. According to Post-Hoc analysis, the mean for age was higher in individuals with two risk alleles (A) than in individuals without risk alleles, GG genotypes. After adjustment for confounders (BMI, age, total energy intake and physical activity) there was significant mean difference for the weight (*p* = 0.03) and BMI (*p* = 0.04), borderline significant difference for economic status (*p* = 0.06) and HC (*p* = 0.06) which post-Hoc analysis showed that their means were lower in individuals with two (A) risk allele than in individuals with GG genotypes (Table 2).

**Table 2:** Characteristics of study population across rs 3807992 genotypes and tertiles of total fat intake

Characteristics	Genotypes						*p* value	*p* value*	Tertile of total fat (g)						*p* value	*p* value*
	GG (n = 117)		AG (n = 51)		AA (n = 53)				T1 (n = 73)		T2 (n = 65)		T3(n = 83)			
	Mean or N	SD or %	Mean or N	SD or %	Mean or N	SD or %			Mean or N	SD or %	Mean or N	SD or %	Mean or N	SD or %		
Age (year)	33.04	9.27	34.31	8.94	37.02^a^	9.81	0.03	0.45	38.68	10.32	33.10	8.46	34.79	9.06	0.002	0.15
Weight (kg)	78.87	11.70	80.66	12.91	81.72^a^	9.43	0.58	0.03	78.70	11.81	76.77	9.70	82.67^a,b^	11.22	0.005	0.91
Height (cm)	160.31	5.58	161.24	5.34	161.38	6.18	0.54	0.16	160.47	6.75	161.60	5.76	161.68	5.37	0.39	0.49
BMI (kg/m^2^)	31.24	2.16	30.92	4.19	31.78	2.66	0.12	0.04	30.50	3.87	29.81	3.47	31.73^a,b^	4.12	0.01	0.86
FM (kg)	35.43	9.70	34.57	8.54	33.50	7.95	0.38	0.28	33.25	8.14	32.91	8.63	36.71^a,b^	9.07	0.01	0.93
Body fat (%)	42.77	6.09	42.50	4.76	41.85	5.30	0.55	0.73	42.11	5.12	41.14	5.40	43.41^b^	5.39	0.03	0.43
FMI (kg/m)	13.77	3.57	13.30	3.07	12.91	3.20	0.29	0.91	13.01	3.12	12.58	3.03	14.09^b^	3.55	0.01	0.66
Arm circumference (cm)	34.30	2.72	33.93	3.87	33.79	2.81	0.74	0.73	33.55	2.55	34.37	3.35	34.67	3.50	0.24	0.66
Physical activity (MET h/wk)	1215.46	2033.81	1379.12	2823.33	1073.74	1761.48	0.74	0.63	1307.73	2529.64	1148.23	1905.40	1154.00	1842.26	0.85	0.45
Marital status	18	26.9	13	19.4	34	50.7	0.63	0.23	32	29.6	35	32.4	41	38	0.41	0.65
Single																
Married	32	20.8	34	22.1	79	51.3			99	35.2	94	33.5	88	31.3		
Economic status	25	45.5	20	36.4	10	18.2	0.03	0.06	20	35.7	19	33.9	17	30.4	0.12	0.07
Poor																
Moderate	55	54.5	17	16.8	29	28.7			29	27.1	35	32.7	43	40.2		
Good	31	59.6	13	25	8	15.4			24	42.9	10	17.9	22	39.3		
Education	1	3.33	1	3.33	1	3.33	0.63	0.07	1	25	1	25	2	50	0.46	0.15
Illiterate																
Under diploma	10	40	8	32	7	28			18	36.7	15	30.6	16	32.7		
Diploma	43	40	22	32	19	28			46	30.5	16	39.7	45	29.8		
Bachelor and higher than	59	60.2	19	19.4	20	20.4			36	34.6	25	41.3	43	24		
Supplement intake	61	56.5	14	15.2	19	20.7	0.10	0.93	66	35.7	53	28.6	66	53.7	0.22	0.41
Yes																
No	113	47.3	47	25.6	31	24			62	35.2	50	28.4	64	36.4		
*Fat distribution*																
WC (cm)	99.73	9.88	98.80	10.70	97.64	9.30	0.43	0.79	97.32	9.17	96.84	9.81	100.83^b^	9.69	0.02	0.84
HC (cm)	105.99	6.38	103.47	16.65	105.20	6.27	0.41	0.06	102.82	13.33	104.54	6.07	107.47^a^	6.67	0.008	0.94
NC (cm)	37.01	2.06	40.88	21.03	36.52	2.52	0.15	0.83	37.23	4.00	36.97	2.60	38.36	11.43	0.47	0.77
WHR (cm)	0.94	0.06	2.86	13.28	0.92	0.05	0.17	0.81	2.17	10.65	0.92	0.05	0.93	0.05	0.36	0.42
WHtR(cm)	0.62	0.06	0.61	0.06	0.60	0.05	0.19	0.72	0.60	0.05	0.60	0.05	0.62^b^	0.05	0.03	0.63
Visceral fat level (cm^2^)	16.08	3.55	16.06	3.18	15.47	3.29	0.43	0.63	15.68	3.33	15.12	3.34	16.56^b^	3.17	0.02	0.78
Right arm fat (kg)	2.75	1.14	2.85	1.29	2.68	0.97	0.78	0.60	2.60	0.98	2.73	1.26	3.05	1.25	0.17	0.90
Right arm fat (%)	297.37	121.9	302.29	131.34	283.37	101.65	0.71	0.93	277.01	103.04	287.63	122.64	322.94	130.74	0.15	0.72
Left arm fat (kg)	2.77	1.12	2.88	1.30	2.71	0.97	0.78	0.48	2.64	0.99	2.75	1.26	3.08	1.26	0.18	0.87
Left arm fat (%)	299.95	121.61	306.43	132.27	286.17	102.13	0.69	0.89	280.69	104.38	289.41	122.27	326.75	131.12	0.14	0.68
Trunk fat (kg)	15.92	3.56	15.96	4.14	15.73	3.29	0.94	0.54	15.38	3.33	15.81	3.87	16.82	3.72	0.14	0.95
Trunk fat (%)	307.08	69.37	303.61	72.82	296.99	63.70	0.76	0.96	292.58	62.35	298.49	66.66	317.79	70.22	0.15	0.64
Left leg fat (kg)	4.95	1.25	5.15	1.54	4.92	1.14	0.72	0.10	4.88	1.23	4.89	1.32	5.31	1.32	0.17	0.70
Left leg fat (%)	210.52	55.80	215.53	60.89	204.70	48.12	0.65	0.56	204.35	52.65	203.30	52.02	221.18	55.01	0.17	0.57
Right leg fat (kg)	4.98	1.28	5.18	1.57	5.18	1.57	0.75	0.10	4.92	1.26	4.92	1.34	5.35	1.34	0.18	0.68
Right leg fat (%)	211.85	56.92	216.81	61.61	206.11	48.53	0.66	0.52	205.75	53.28	204.68	52.57	222.68	56.05	0.18	0.57
*Biochemical parameters*																
FBS (mg/dl)	85.07	9.16	84.37	7.04	88.44	9.24	0.13	0.60	88.17	9.33	84.50	7.18	88.19	9.91	0.14	0.62
CHOL (mg/dl)	171.34	30.85	166.68	27.28	179.45	31.76	0.25	0.34	174.37	30.33	177.61	37.43	175.78	26.69	0.92	0.59
TG (mg/dl)	140.03	80.39	103.37	50.18	109.15	51.17	0.06	0.10	122.20	69.21	117.67	68.26	108.51	45.81	0.62	0.19
HDL-C (mg/dl)	46.61	8.62	46.06	6.99	47.76	10.37	0.76	0.52	44.48	9.30	50.38^a^	10.42	46.63	7.84	0.03	0.10
LDL-C (mg/dl)	92.65	21.21	91.06	21.65	98.89	22.13	0.29	0.49	96.34	20.19	96.52	26.80	96.65	19.57	0.99	0.81
LDL/HDL	2.02	0.48	2.04	0.63	2.14	0.59	0.62	0.73	2.23	0.56	1.96	0.61	2.12	0.52	0.17	0.38
CHOL/HDL	3.75	0.76	3.71	0.89	3.87	0.86	0.72	0.87	4.03	0.88	3.61	0.86	3.85	0.75	0.13	0.16
Insulin (mIU/ml)	1.21	0.02	1.22	0.03	1.22	0.02	0.87	0.22	1.20	0.20	1.21	0.22	1.22	0.25	0.70	0.88
HOMA IR	2.94	0.98	2.53	1.00	3.20	1.15	0.10	0.41	3.25	1.10	2.85	1.20	3.02	1.00	0.37	0.71

The use of chi-square, t-test, and ANOVA test

All data are presented as mean, SD or N and %

*p* value * obtained from ANCOVA test adjusted for age, BMI, energy intake, and physical activity

*p* value < 0.05 was considered significant

*BMI* body mass index, *FM* fat mass, *FMI* fat mass index, *WC* waist circumference, *HC* hip circumference, *NC* neck circumference, *WHR* weight to hip ratio, *WHtR* weight to height ratio, *FBS* fasting blood sugar, *CHOL* cholesterol, *TG* triglyceride, *HDL-C* high-density lipoprotein-cholesterol, *LDL-C* low-density lipoprotein-cholesterol, *HOMA IR* homeostatic model assessment of insulin resistance

^a^Significant compared to tertile 1 or GG genotype

^b^Significance compared to tertile 2

### Association between anthropometric measurements, body fat distribution, and biochemical parameters among tertiles of total fat

There were significant mean difference for age (*p* = 0.002), weight (*p* = 0.005), BMI (*p* = 0.01), FM (*p* = 0.01), percent body fat (*p* = 0.03), FMI (*p* = 0.01), WC (*p* = 0.02), HC (*p* = 0.008), WHtR (*p* = 0.03), visceral fat level (*p* = 0.02), serum HDL-C level (*p* = 0.03) in the tertiles of total fat in the crude model. In post-hoc analysis (Bonferroni), we found significant differences for means WC, WHtR, visceral fat level, FMI, percent body fat in tertile 2 and tertile 3 and also there was significant difference for the mean of HC in tertile 1 and 3, serum HDL-C in tertiles 1 and 2, that their mean in tertile 3 was higher than tertile 2 and tertile 1 and also there was significant difference for mean weight, BMI and body fat mass in between tertiles 3 and 1, tertiles 3 and 2, and their mean was higher in tertile 3 (Table 2).

### Association between anthropometric measurements, body fat distribution, and biochemical parameters among tertiles of SFA

In the crude model, there were significant mean differences for age (*p* = 0.04), HC (*p* = 0.01), and borderline significant difference for FM (*p* = 0.06) across tertiles of SFA. No significant differences were found for other variables. Post-Hoc analysis (Bonferroni) showed that the mean HC in tertile 3 was higher than tertile 1. After adjusting for potential confounder (age, physical activity, energy intake and BMI), there were significant mean differences in physical activity (*p* = 0.02). Before adjusting for confounders, no significant difference was found between SFA and biochemical parameters (*p* > 0.05). After adjusting for confounder, there were significant mean differences for insulin resistance (*p* = 0.04) across tertiles of SFA (Table 3).

**Table 3:** Study participant characteristics between tertile all type fats with anthropometric indices, fat distribution, and biochemical variables

Characteristics	SFA (g)						*p* value	*p* value*	TFA (g)						*p* value	*p* value*
	T1 (n = 70)		T2 (n = 74)		T3 (n = 77)				T1 (n = 110)		T2 (n = 37)		T3 (n = 74)			
	Mean	SD	Mean	SD	Mean	SD			Mean	SD	Mean	SD	Mean	SD		
Age (year)	37.65	10.58	35.50	8.13	33.77^a^	9.61	**0.04**	0.29	35.24	10.22	35.67	8.95	36.04	8.94	0.85	0.66
Weight (kg)	78.74	11.88	78.39	10.98	81.64	10.73	0.16	0.33	80.44	11.20	82.77	12.33	76.91^b^	10.28	**0.02**	0.40
Height (cm)	160.87	6.63	160.97	5.91	161.88	5.38	0.52	0.73	161.33	5.83	161.85	6.49	160.87^b^	5.95	0.71	**0.02**
physical activity (METh/wk)	1289.06	1659.24	883.52	844.43	887.97	780.69	0.22	**0.02**	937.07	939.23	931.96	959.04	1047.31	1392.65	0.92	0.93
BMI (kg/${m}^{2}$)	30.37	3.94	30.51	3.72	31.36	4.07	0.25	0.17	31.01	3.91	31.86	4.26	29.87^b^	3.62	**0.03**	0.66
FM (kg)	33.34	8.33	33.52	8.53	36.35	9.21	**0.06**	0.30	34.97	8.85	37.28	9.30	32.27^b^	7.97	**0.01**	0.70
body fat (%)	42.18	5.19	41.50	5.03	43.21	5.76	0.14	0.30	42.62	5.46	43.43	5.68	41.29	4.95	0.09	0.93
FMI (kg/m)	12.92	3.13	13.91	3.54	13.92	3.31	0.12	0.43	13.51	3.32	14.21	3.68	12.50^b^	2.98	**0.02**	0.62
Arm circumference (cm)	33.29	2.73	34.68	3.36	34.31	3.65	0.12	0.24	34.31	3.42	35.60	4.02	33.37	2.26	**0.02**	0.98
*Fat distribution*																
WC (cm)	97.54	9.47	97.67	9.83	100.17	9.64	0.17	0.30	99.37	10.01	101.10	10.88	95.91^a,b^	7.98	**0.01**	0.58
HC (cm)	102.54	13.53	105.51	6.29	106.95 ^a^	6.71	**0.01**	0.08	106.04	6.37	104.76	18.96	103.78	5.53	0.28	0.32
NC (cm)	36.22	2.24	37.02	2.40	39.03	14.92	0.34	0.14	38.36	12.61	37.00	2.54	36.55	2.49	0.09	0.12
WHtR (cm)	0.60	0.05	0.60	0.05	0.61	0.05	0.36	0.84	0.61	0.05	0.62	0.06	0.59^a,b^	0.04	**0.02**	0.17
WHR (cm)	2.23	10.88	0.92	0.05	0.93	0.05	0.33	0.69	0.93	0.05	3.39	14.97	0.92	0.04	0.08	0.30
Visceral fat level (cm^2^)	15.71	3.43	15.32	3.27	16.45	3.19	0.10	0.46	16.18	3.36	16.62	3.26	14.95^a,b^	3.12	**0.01**	0.92
Right arm fat (kg)	2.54	0.99	2.94	1.33	2.92	1.18	0.23	0.26	2.93	1.31	3.33	1.45	2.47^b^	0.76	**0.01**	0.63
Right arm fat (%)	271.76	104.99	310.56	132.32	308.53	121.38	0.26	0.33	30.55	133.45	34.07	147.86	26.30^b^	78.15	**0.02**	0.85
Left arm fat (kg)	2.58	0.98	2.96	1.32	2.95	1.19	0.25	0.27	2.95	1.31	3.35	1.44	2.50^b^	0.76	**0.01**	0.42
Left arm fat (%)	274.91	105.78	313.11	131.99	312.35	122.54	0.27	0.40	31.12	134.03	34.01	148.78	26.36^b^	78.14	**0.02**	0.74
Trunk fat (kg)	15.13	3.40	16.52	3.97	16.44	3.52	0.15	0.44	16.32	3.97	17.60	4.14	15.17^b^	2.81	**0.03**	0.90
Trunk fat (%)	288.60	65.37	312.54	69.75	309.09	65.56	0.21	0.53	30.81	71.64	32.85	76.62	28.70	53.98	0.08	0.77
Right leg fat (kg)	4.80	1.19	5.15	1.41	5.26	1.33	0.26	0.35	5.20	1.40	5.72	1.58	4.68^b^	0.94	**0.007**	0.58
Right leg fat (%)	201.53	49.95	214.67	56.31	218.57	55.84	0.33	0.44	21.88	56.81	23.43	68.33	19.64^b^	39.25	**0.01**	0.66
Left leg fat (kg)	4.77	1.16	5.12	1.39	5.23	1.30	0.25	0.31	5.15	1.37	5.67	1.56	4.66^b^	0.94	**0.008**	0.58
Left leg fat (%)	200.26	49.42	213.17	55.62	217.08	54.80	0.33	0.42	21.15	55.79	23.82	67.93	19.69^b^	38.82	**0.01**	0.68
*Biochemical parameters*																
FBS (mg/dl)	88.10	9.78	85.56	7.20	87.73	10.27	0.43	0.37	86.89	9.42	87.25	5.54	87.00	9.48	0.99	0.68
CHOL (mg/dl)	177.93	32.26	177.48	33.83	174.94	27.84	0.92	0.78	177.85	34.31	170.41	26.12	175.41	29.32	0.75	0.76
TG (mg/dl)	107.10	41.14	123.34	76.78	112.67	51.42	0.52	0.66	112.61	63.53	101.50	53.56	122.27	58.81	0.52	0.90
HDL-C (mg/dl)	47.00	10.38	48.21	9.80	46.32	7.99	0.67	0.78	48.38	10.39	47.83	7.42	45.81	8.58	0.41	0.94
LDL-C (mg/dl)	98.72	22.88	95.97	23.15	95.32	20.69	0.81	0.14	97.48	22.82	94.25	20.67	96.06	22.16	0.89	0.50
LDL/HDL	2.16	0.55	2.05	0.60	2.11	0.55	0.73	0.84	2.06	0.51	2.05	0.72	2.16	0.58	0.70	0.73
CHOL/HDL	3.88	0.79	3.74	0.90	3.86	0.79	0.73	0.27	3.76	0.77	3.69	1.07	3.93	0.83	0.52	0.91
Insulin (mIU/ml)	1.17	0.19	1.24	0.23	1.22	0.24	0.78	**0.04**	1.20	0.23	1.22	0.21	1.22	0.23	0.78	0.86
HOMA IR	3.03	1.18	3.02	0.87	3.04	1.28	0.99	0.98	2.94	1.07	2.80	1.13	3.19	1.12	0.44	0.55

Characteristics	MUFA (g)						*p* value	*p* value*	PUFA (g)						*p* value	*p* value*
	T1 (n = 69)		T2 (n = 68)		T3 (n = 84)				T1 (n = 73)		T2 (n = 63)		T3 (n = 85)			
	Mean	SD	Mean	SD	Mean	SD			Mean	SD	Mean	SD	Mean	SD		
Age (year)	32.27	10.73	34.73	8.37	34.88	9.39	0.20	0.32	36.94	10.59	35.55	8.97	34.43	9.01	0.26	0.52
Weight (kg)	78.98	12.36	77.28	9.71	82.13^b^	11.07	**0.02**	0.97	79.86	11.65	77.07	10.79	81.34^b^	10.99	0.07	0.60
Height (cm)	160.66	6.87	161.49	5.30	161.26	5.97	0.61	0.55	161.04	6.50	161.24	5.81	161.45	5.65	0.91	0.33
physical activity (METh/wk)	1146.95	1480.10	995.48	1096.42	878.16	758.12	0.56	0.28	814.27	817.43	1046.54	1579.41	1067.58	822.20	0.57	0.61
BMI (kg/${m}^{2}$)	30.71	4.10	29.60	3.32	31.76^b^	4.00	**0.003**	0.53	30.94	3.91	29.68	3.52	31.41^b^	4.04	**0.02**	0.61
FM (kg)	33.84	8.58	32.00	7.49	36.94^a,b^	9.35	**0.002**	0.70	34.58	8.51	32.26	8.45	35.96^b^	9.01	**0.03**	0.82
body fat (%)	42.42	4.99	40.93	5.10	43.34^b^	5.68	**0.02**	0.92	42.52	5.09	41.06	5.55	43.05^b^	5.36	0.07	0.56
FMI (kg/m)	13.19	3.19	12.30	2.82	14.16^b^	3.57	**0.002**	0.40	13.41	3.11	12.43	3.10	13.83^b^	3.53	**0.03**	0.63
Arm circumference (cm)	33.70	2.73	33.33	2.58	35.17	3.74	**0.01**	0.33	33.98	2.67	33.44	3.14	34.88^b^	3.50	0.07	0.56
*Fat distribution*																
WC (cm)	97.81	9.62	96.27	9.18	100.91^b^	9.73	**0.01**	0.65	99.35	9.37	95.29^a^	9.19	100.93^b^	9.58	**0.007**	0.46
HC (cm)	103.09	13.86	104.14	5.86	107.49^b^	6.49	**0.01**	0.59	103.66	13.61	104.24	6.01	106.90^a^	6.57	0.07	0.14
NC (cm)	36.69	2.72	36.26	2.49	38.83	13.54	0.35	0.45	36.70	2.44	36.45	2.59	38.98	13.53	0.28	0.71
WHtR (cm)	0.60	0.05	0.59	0.05	0.62	0.06	**0.007**	0.32	0.61	0.05	0.59^a^	0.05	0.62^b^	0.06	**0.006**	0.70
WHR (cm)	2.25	10.96	0.92	0.05	0.94	0.05	0.33	0.66	2.19	10.65	0.91	0.05	0.93	0.05	0.35	0.32
Visceral fat level (cm^2^)	15.85	3.35	15.01	3.39	16.51^b^	3.10	**0.02**	0.75	16.28	3.15	14.82^a^	3.63	16.22^b^	3.08	**0.01**	0.80
Right arm fat (kg)	2.78	1.11	2.49	0.93	3.12^b^	1.37	**0.03**	0.64	2.86	1.06	2.57	1.19	3.01	1.26	0.17	0.75
Right arm fat (%)	293.69	113.02	265.29	98.35	329.93^b^	138.01	**0.02**	0.47	300.43	109.30	272.61	117.79	319.57	138.01130.52	0.15	0.59
Left arm fat (kg)	2.81	1.11	2.51	0.92	3.16^b^	1.37	**0.02**	0.66	2.89	1.08	2.60	1.18	3.04	1.26	0.18	0.80
Left arm fat (%)	297.01	114.18	267.80	98.43	333.45^b^	138.05	**0.02**	0.44	303.41	111.22	275.92	117.57	322.67	130.67	0.16	0.62
Trunk fat (kg)	15.87	3.65	15.08	3.12	17.09^b^	3.95	**0.02**	0.53	16.30	3.41	15.09	3.82	16.79^b^	3.64	**0.06**	0.55
Trunk fat (%)	300.46	65.85	286.47	58.41	322.20^b^	71.92	**0.02**	0.31	306.91	62.32	286.70	67.26	317.84^b^	68.63	**0.06**	0.62
Right leg fat (kg)	5.08	1.29	4.71	1.21	5.40^b^	1.38	**0.03**	0.84	5.10	1.24	4.85	1.37	5.28	1.34	0.27	0.86
Right leg fat (%)	211.79	51.20	197.02	51.94	224.96^b^	56.43	**0.03**	0.57	211.23	50.39	203.12	55.80	220.04	55.70	0.28	0.61
Left leg fat (kg)	5.05	1.26	4.68	1.20	5.37^b^	1.35	**0.03**	0.82	5.06	1.22	4.82	1.35	5.24	1.31	0.27	0.86
Left leg fat (%)	210.26	50.58	195.80	51.52	223.42^b^	55.32	**0.03**	0.56	209.75	49.77	201.95	55.33	218.83	54.62	0.29	0.60
*Biochemical parameters*																
FBS (mg/dl)	86.64	9.41	86.50	7.64	87.66	9.95	0.83	0.96	85.93	8.17	86.94	8.79	87.77	9.90	0.70	0.96
CHOL (mg/dl)	176.48	33.27	173.52	32.94	177.74	32.54	0.84	0.57	176.55	33.15	175.74	28.99	175.80	28.71	0.99	0.17
TG (mg/dl)	113.80	55.10	124.24	78.62	108.76	44.66	0.54	0.74	109.82	64.98	121.97	78.62	113.50	46.72	0.70	0.43
HDL-C (mg/dl)	47.32	10.59	47.14	9.82	47.30	8.09	0.99	0.93	47.17	10.28	46.97	9.63	47.57	8.64	0.96	0.40
LDL-C (mg/dl)	96.12	22.69	95.14	22.84	98.05	21.53	0.85	0.47	96.65	22.37	95.14	20.31	97.65	23.90	0.88	0.54
LDL/HDL	2.10	0.58	2.07	0.56	2.13	0.57	0.92	0.94	2.11	0.58	2.07	0.48	2.12	0.64	0.91	0.57
CHOL/HDL	3.83	0.82	3.78	0.89	3.84	0.80	0.94	0.17	3.85	0.87	3.82	0.71	3.80^a^	0.91	0.97	**0.05**
Insulin (mIU/ml)	1.18	0.21	1.21	0.20	1.25	0.27	0.20	0.27	1.20	0.20	1.22	0.22	1.21	0.25	0.82	0.99
HOMA IR	3.19	1.14	2.84	1.18	3.06	0.98	0.45	0.78	3.05	1.10	3.02	1.25	3.03	0.97	0.99	0.84

All data are presented as mean and SD

*p* value obtained from the ANOVA test

*p* value < 0.05 was considered significant. P-value < 0.05 were bolded

*p* value * obtained from ANCOVA test adjusted for age, BMI, energy intake, and physical activity

*SFA* saturated fatty acid, *TFA* trans-fatty acid, *MUFA* monounsaturated fatty acid, *PUFA* polyunsaturated fatty acid, *BMI* body mass index, *FM* fat mass, *FMI* fat mass index, *WC* waist circumference, *HC* hip circumference, NC: neck circumference, WHtR: weight to height ratio, *WHR* weight to hip ratio, *FBS* fasting blood sugar, *CHOL* cholesterol, *TG* triglyceride, *HDL-C* high-density lipoprotein-cholesterol, *LDL-C* low-density lipoprotein-cholesterol, *HOMA IR* homeostatic model assessment of insulin resistance

^a^Significant compared to tertile 1

^b^Significance compared to tertile 2

### Association between anthropometric measurements, body fat distribution, and biochemical parameters among tertile of TFA

There were significant difference for weight (*p* = 0.02), BMI (*p* = 0.03), FM (*p* = 0.01), FMI (*p* = 0.02), arm circumference (*p* = 0.02), WC (*p* = 0.01), WHtR (*p* = 0.02), visceral fat level (*p* = 0.01), right arm fat (*p* = 0.01) and its percentage (*p* = 0.02), left arm fat (*p* = 0.01) and its percentage (*p* = 0.02), trunk fat (*p* = 0.03), right leg fat (*p* = 0.007) and and its percentage (*p* = 0.01), left leg fat (*p* = 0.008) and and its percentage (*p* = 0.01) across tertiles of TFA in crude model. According to Post-Hoc (Bonferroni) analysis, there was significant difference for means weight, BMI, body fat mass, arm circumference, WHtR, FMI, right arm fat, and its percentage, left arm fat and its percentage, trunk fat, right leg fat, and its percentage, left leg fat and its percentage in tertile 3 and tertile 2 and also significant difference for mean visceral fat in tertile 3 and 2 and tertile 3 and 1, and their mean was lower in tertile 3. After adjusting for confounder (age, physical activity, energy intake and BMI), there was significant difference for mean height (*p* = 0.02) which means lower in tertile 3 and no significant difference was found between TFA and fat distribution pattern indices (*p* > 0.05). Before and after adjusting for confounder, no significant difference was found between TFA and biochemical parameters (*p* > 0.05) (Table 3).

### Association between anthropometric measurements, body fat distribution and biochemical parameters among tertile of MUFA intake

We found significant difference for weight (*p* = 0.02), BMI (*p* = 0.003), FM (*p* = 0.002), body fat percentage (*p* = 0.02), FMI (*p* = 0.002), arm circumference (*p* = 0.01), WC (*p* = 0.01), HC (*p* = 0.01), WHtR (*p* = 0.007), visceral fat level (*p* = 0.02), right arm fat (*p* = 0.03) and its percentage (*p* = 0.02), left arm fat (*p* = 0.02) and its percentage (*p* = 0.02), trunk fat (*p* = 0.02) and its percentage (*p* = 0.02), right leg fat (*p* = 0.03) and its percentage (*p* = 0.03) and left leg fat (*p* = 0.03) and its percentage, across tertiles of MUFA in the crude model. Post-Hoc analysis (Bonferroni) showed that the mean body weight, WC, visceral fat level, FMI, fat right arm and its percentage, fat left arm and its percentage, trunk fat and its percentage, fat right leg and its percentage, and fat left leg and its percentage in tertile 3 was higher than tertile 1 and the mean FM in tertile 3 was higher than tertile 1and 2. After adjusting for confounder (age, physical activity, energy intake and BMI), no significant difference was found between MUFA and fat distribution pattern indices (*p* > 0.05). Before and after adjusting for confounder, no significant diffrence was found between MUFA and biochemical parameters (*p* > 0.05) (Table 3).

### Association between anthropometric measurements, body fat distribution, and biochemical parameters among tertile PUFA intake

In the crude model, there were significant difference for BMI (*p* = 0.02), FM (*p* = 0.03), FMI (*p* = 0.03), WC (*p* = 0.007), WHtR (*p* = 0.006), visceral fat level (*p* = 0.01) and borderline significant difference for fat in the trunk fat (*p* = 0.06) and its percentage (*p* = 0.06) across tertiles of PUFA.Post-Hoc analysis (Bonferroni) showed that the mean BMI, FMI, FM, trunk fat, and its percentage in tertile 2 was lower than tertile 3, and the mean visceral fat level, WC, and WHtR in tertile 2 was lower than tertile 1 and 3. After adjusting for confounder (age, physical activity, energy intake and BMI), no significant difference was found between PUFA and fat distribution pattern indices (*p* > 0.05). We found no significant difference between PUFA and biochemical parameters, before adjustment (*p* > 0.05). There was borderline significant difference for CHOL/HDL (*p* = 0.05) across tertiles of PUFA, after adjustment. Post-Hoc analysis (Bonferroni) showed that the mean CHOL/HDL in tertile 3 was higher than tertile 1 (Table 3).

### Association between anthropometric measurements, body fat distribution, and biochemical parameters among tertile linoleic acid

There were significant differences for WC (*p* = 0.01) and WHtR (*p* = 0.02), there were borderline significant differences for BMI (*p* = 0.05), FM (*p* = 0.05), visceral fat level (*p* = 0.06), across tertiles of linoleic acid in the crude model. No significant differences were observed for other variables. Post-Hoc analysis (Bonferroni) showed that the mean BMI. FM, WC, and WHtR and visceral fat levels in tertile 3 were higher than tertile 2. After adjusting for confounder (age, physical activity, energy intake and BMI), no significant difference was found between linoleic acid and fat distribution pattern indices (*p* > 0.05). We found no significant difference between linoleic acid and biochemical parameters, before adjustment (*p* > 0.05). There was a borderline significant difference for CHOL/HDL (*p* = 0.06) after adjustment post-hoc analysis (Bonferroni) showed that the mean CHOL/HDL in tertile 3 was higher than tertile 1 (Table 4).

**Table 4:** Study participant characteristics between tertiles of all type of fats with anthropometric indices, fat distribution and biochemical variables

Characteristics	linoleic acid (g)						*p* value	*p* value*	ALA (g)			
	T1 (n = 73)		T2 (n = 64)		T3 (n = 84)				T1 (n = 81)		T2 (n = 66)	
	Mean	SD	Mean	SD	Mean	SD			Mean	SD	Mean	SD
Age (year)	37.01	10.62	35.73	8.97	34.22	8.95	0.18	0.40	36.88	9.99	35.59	9.12
Weight (kg)	79.87	11.65	77.07	10.65	81.39	11.09	0.07	0.36	80.01	11.73	78.76	10.18
Height (cm)	161.33	6.58	160.82	5.67	161.52	5.67	0.76	0.24	161.41	6.76	161.51	4.97
physical activity(METh/wk)	885.95	845.60	934.65	1427.46	1108.98	1000.2	0.63	0.69	721.67	735.89	1031.72	1267.96
BMI (kg/${\mathrm{m}}^{2}$)	30.84	3.95	29.84	3.59	31.40 ^b^	4.04	**0.05**	0.59	30.74	3.72	30.37	3.75
FM (kg)	34.41	8.61	32.50	8.29	35.97 ^b^	9.09	**0.05**	0.80	34.26	8.45	33.42	7.76
body fat (%)	42.30	5.28	41.39	5.32	43.02	5.43	0.19	0.61	42.12	5.12	41.76	5.26
FMI (kg/m)	13.30	3.18	12.58	3.06	13.81	3.54	0.08	0.77	13.21	3.16	12.86	2.91
Arm circumference (cm)	33.91	2.66	33.62	3.27	34.78	3.47	0.33	0.53	33.86	3.09	33.71	2.56
*Fat distribution*												
WC (cm)	99.14	9.49	95.67	9.05	100.10 ^b^	9.96	**0.01**	0.34	99.03	9.60	97.87	9.37
HC (cm)	103.64	13.60	104.29	6.06	106.91	6.55	0.07	0.39	104.01	13.09	105.12	6.00
NC (cm)	36.60	2.62	36.37	2.48	38.95	3.53	0.29	0.55	36.83	2.32	36.41	2.35
WHR (cm)	2.18	10.65	0.91	0.05	0.93	0.05	0.35	0.33	2.06	10.11	0.92	0.05
WHtR (cm)	0.61	0.05	0.59	0.05	0.62	0.06	**0.02**	0.78	0.61	0.05	0.60	0.05
Visceral fat level (cm^2^)	16.13	3.28	15.03	3.46	16.21 ^b^	3.16	**0.06**	0.75	15.92	3.19	15.66	3.29
Right arm fat (kg)	2.82	1.07	2.61	1.19	3.00	1.27	0.27	0.77	2.67	1.03	2.70	0.99
Right arm fat (%)	295.56	111.22	278.55	118.36	317.63	130.37	0.27	0.75	284.34	110.27	285.12	99.95
Left arm fat (kg)	2.85	1.09	2.64	1.18	3.03	1.27	0.27	0.83	2.70	1.04	2.72	0.97
Left arm fat (%)	298.57	112.94	282.04	118.55	320.57	130.32	0.29	0.78	287.55	111.78	287.03	98.85
Trunk fat (kg)	16.13	3.46	15.30	3.81	16.72	3.69	0.16	0.47	15.67	3.48	15.81	3.23
Trunk fat (%)	303.02	64.51	292.13	67.16	315.78	68.86	0.21	0.82	297.99	66.89	297.84	59.42
Right leg fat (kg)	5.08	1.25	4.88	1.37	5.27	1.34	0.35	0.94	4.89	1.23	5.01	1.19
Right leg fat (%)	209.79	51.05	205.40	56.25	219.47	55.42	0.41	0.79	204.69	52.56	207.54	48.12
Left leg fat (kg)	5.04	1.23	4.85	1.35	5.23	1.32	0.36	0.95	4.85	1.21	4.98	1.18
Left leg fat (%)	208.36	50.43	204.11	55.64	217.91	54.44	0.42	0.79	203.21	52.10	206.40	47.65
*Biochemical parameters*												
FBS (mg/dl)	86.31	8.23	87.70	9.93	86.85	8.93	0.82	0.69	88.70	8.46	86.19	8.30
CHOL (mg/dl)	175.24	32.61	178.52	28.70	174.41	32.92	0.84	0.15	178.23	32.12	175.41	28.48
TG (mg/dl)	110.06	65.02	119.85	63.15	115.29	55.64	0.81	0.69	119.11	67.12	124.93	65.92
HDL-C (mg/dl)	47.24	10.28	47.50	9.33	47.07	8.95	0.98	0.77	47.58	10.64	45.93	7.27
LDL-C (mg/dl)	94.96	21.68	97.20	19.62	97.03	24.73	0.90	0.99	99.02	21.88	97.70	21.98
LDL/HDL	2.07	0.57	2.09	0.48	2.13	0.64	0.92	0.74	2.15	0.57	2.16	0.57
CHOL/HDL	3.81	0.86	3.83	0.71	3.81 ^a^	0.92	0.99	**0.06**	3.87	0.76	3.72	0.83
Insulin (mIU/ml)	1.20	0.20	1.21	0.21	1.23	0.26	0.69	0.64	1.21	0.20	1.20	0.23
HOMA IR	3.06	1.09	3.10	1.29	2.95	0.94	0.84	0.58	3.26	1.14	2.90	1.17

Characteristics	ALA (g)		*p* value	*p* value*	EPA and DHA (g)						*p* value	*p* value*
	T3 (n = 74)				T1 (n = 78)		T2 (n = 86)		T3 (n = 57)			
	Mean	SD			Mean	SD	Mean	SD	Mean	SD		
Age (year)	34.14	9.40	0.20	0.60	36.05	9.53	36.30	10.31	33.85	8.32	0.28	0.94
Weight (kg)	79.97	11.71	0.76	0.99	78.76	10.75	79.56	11.36	80.89	11.79	0.56	0.43
Height (cm)	160.85	5.91	0.75	0.39	160.48	5.50	161.88	6.43	161.37	5.84	0.32	0.99
physical activity(METh/wk)	1197.83	1229.15	0.14	0.09	1016.05	1111.52	824.10	706.16	1157.59	1431.72	0.41	0.44
BMI (kg/${\mathrm{m}}^{2}$)	31.14	4.29	0.51	0.60	30.73	3.88	30.46	3.75	31.24	4.22	0.51	0.63
FM (kg)	35.59	9.91	0.33	0.34	34.52	8.26	34.06	9.10	34.95	9.12	0.83	0.95
body fat (%)	43.01	5.72	0.36	0.18	42.79	5.04	41.83	5.56	42.38	5.53	0.51	0.66
FMI (kg/m)	13.75	3.77	0.40	0.22	13.42	3.18	13.05	3.34	13.47	3.48	0.69	0.93
Arm circumference (cm)	34.78	3.68	0.22	0.23	33.86	3.07	34.21	3.39	34.42	3.18	0.74	0.51
*Fat distribution*												
WC (cm)	98.48	10.16	0.77	0.55	98.16	9.61	98.77	10.06	98.55	9.35	0.92	0.40
HC (cm)	106.21	6.85	0.36	0.44	105.29	6.10	105.36	6.59	104.33	15.30	0.79	0.67
NC (cm)	38.87	14.16	0.37	0.39	38.86	16.10	36.92	2.51	36.90	2.22	0.52	0.61
WHR (cm)	0.92	0.06	0.41	0.35	0.93	0.06	0.93	0.05	2.52	12.06	0.24	0.29
WHtR (cm)	0.61	0.06	0.66	0.48	0.61	0.05	0.61	0.05	0.61	0.05	0.98	0.78
Visceral fat level (cm^2^)	15.91	3.50	0.87	0.25	16.05	3.05	15.62	3.51	15.89	3.40	0.71	0.94
Right arm fat (kg)	3.05	1.43	0.21	0.33	2.74	1.20	2.80	1.22	2.95	1.17	0.72	0.96
Right arm fat (%)	322.01	142.39	0.21	0.28	291.96	117.54	294.62	123.06	312.69	126.42	0.70	0.98
Left arm fat (kg)	3.08	1.43	0.20	0.29	2.76	1.20	2.83	1.22	2.97	1.19	0.72	0.98
Left arm fat (%)	325.91	142.59	0.19	0.25	294.81	116.90	297.64	123.26	316.27	128.08	0.68	0.95
Trunk fat (kg)	16.70	4.13	0.33	0.29	15.70	3.46	16.10	3.91	16.51	3.65	0.62	0.87
Trunk fat (%)	315.54	72.66	0.33	0.18	299.59	56.26	302.46	71.41	219.25	58.90	0.63	0.77
Right leg fat (kg)	5.33	1.48	0.23	0.34	5.09	1.28	4.98	1.34	5.26	1.37	0.59	0.80
Right leg fat (%)	222.51	59.50	0.21	0.24	214.55	49.83	205.92	54.68	219.25	58.90	0.47	0.85
Left leg fat (kg)	5.30	1.45	0.21	0.35	5.07	1.25	4.94	1.32	5.22	1.34	0.57	0.77
Left leg fat (%)	220.93	58.30	0.21	0.23	213.18	48.85	204.49	54.26	217.76	57.80	0.46	0.84
*Biochemical parameters*												
FBS (mg/dl)	86.10	9.99	0.40	0.26	86.80	8.20	87.83	9.54	85.93	9.22	0.67	0.27
CHOL (mg/dl)	174.48	32.25	0.87	0.16	171.74	26.77	179.34	33.61	175.56	32.52	0.58	0.63
TG (mg/dl)	104.38	48.20	0.33	0.58	107.61	59.54	103.27	38.85	140.56 ^a, b^	78.43	**0.02**	0.47
HDL-C (mg/dl)	48.02	9.78	0.63	0.32	46.61	10.11	49.04	9.74	45.36	7.72	0.23	0.99
LDL-C (mg/dl)	93.41	22.65	0.52	0.88	92.00	19.18	100.83	22.47	95.03	23.98	0.21	0.58
LDL/HDL	2.00	0.56	0.39	0.16	2.06	0.61	2.11	0.57	2.12	0.52	0.91	0.69
CHOL/HDL	3.82	0.83	0.67	**0.03**	3.83	0.94	3.74	0.82	3.92	0.74	0.64	0.74
Insulin (mIU/ml)	1.22	0.24	0.74	0.90	1.19	0.22	1.24	0.24	1.20	0.21	0.27	0.49
HOMA IR	2.92	0.98	0.34	0.25	2.92	0.96	3.00	1.10	3.19	$1$.24	0.63	0.74

All data are presented as mean and SD

*p* value obtained from the ANOVA test

*p* value * obtained from ANCOVA test adjusted for age, BMI, energy intake, and physical activity. P-value < 0.05 were bolded

*ALA* alpha-linolenic acid, *EPA-DHA* eicosapentaenoic acid and docosahexaenoic acid, *BMI* body mass index, *FM* fat mass, *FMI* fat mass index, *WC* waist circumference, *HC* hip circumference, *NC* neck circumference, *WHR* weight to hip ratio, *WHtR* weight to height ratio, *FBS* fasting blood sugar, *CHOL* cholesterol, *TG* triglyceride, *HDL-C* high-density lipoprotein-cholesterol, *LDL-C* low-density lipoprotein-cholesterol, *HOMA IR* homeostatic model assessment of insulin resistance

^a^Significant compared to tertile 1

^b^Significance compared to tertile 2

### Association between anthropometric measurements, body fat distribution, and biochemical parameters with tertile ALA

Before and after adjusting confounder variables (age, BMI, physical activity and energy intake), no significant difference was found between ALA and fat distribution pattern indices (*p* > 0.05).

In the crude model, no significant difference was found between ALA acid and biochemical parameters (*p* > 0.05). There was significant difference for CHOL/HDL (*p* = 0.03) across tertiles of ALA, after adjustment. No significant differences were observed for other variables. Post-Hoc analysis (Bonferroni) showed that the mean CHOL/HDL in tertile 3 was lower than tertile 1 (Table 4).

### Association between anthropometric measurements, body fat distribution and biochemical parameters among tertile of EPA and DHA

Before and after adjustment with potential confounder variables (age, BMI, physical activity, energy intake), there was no significant difference for body fat distribution pattern indices (*p* > 0.05).

We found significant difference for TG (*p* = 0.02), in the crude model. Post-Hoc analysis (Bonferroni) showed that the mean TG in tertile 3 was lower than tertile 1 and 2. No significant differences were observed for other variables (Table 4).

### Interaction between total fat intake with *CAV-1* genotypes on fat distribution variables

By use of the generalized linear model test, the interaction between *rs 3807992* and total fat intake on fat distribution variables was examined. In the crude model, there was positive borderline significant interaction between total fat intake and AG genotype in comparison with the reference group (GG) on visceral fat level (β: 0.17; CI − 0.05, 0.00; *p* value: 0.05) (Table 5).

**Table 5:** Interaction between Caveolin 1 genotypes and dietary fat types with fat distribution

	FM (kg)		*p* value	Visceral fat level (cm^2^)		*p* value	Trunk fat (kg)		*p* value	WC (cm)		*p* value
	B	CI		B	CI		B	CI		B	CI	
*Crude*												
Total fat*AA	0.02	− 0.96, 0.13	0.73	0.11	− 0.01, 0.04	0.86	0.02	− 0.00, 0.05	0.76	0.09	− 0.00, 0.18	0.19
Total fat*AG	− 0.00	− 0.12, 0.10	0.90	0.17	− 0.05, 0.00	0.05	0.00	0.05, 0.06	0.83	0.02	− 0.10, 0.15	0.66
*Adjusted*												
Total fat*AA	0.56	− 4.36, 5.5	0.82	14.78	5.71, 23.78	0.001	8.53	6.20, − 3.61	0.01	0.00	− 0.06, 0.08	0.05
Total fat*AG	1.73	− 4.27, 7.74	0.57	7.53	− 7.20, 21.90	0.05	3.00	7.76, − 12.22	0.14	0.07	− 0.00, 0.14	0.08
*Crude*												
SFA*AA	0.12	− 0.00, 0.18	0.15	0.04	0.08, 1.13	0.33	0.05	− 0.03, 0.14	0.23	0.17	− 0.02,0.36	0.04
SFA*AG	0.06	0.08, 0.04	0.53	0.03	0.12, 0.01	0.28	− 0.04	− 0.15, 0.06	0.41	0.17	0.40,0.04	0.15
*Adjusted*												
SFA*AA	0.009	− 0.01, 0.05	0.72	0.05	− 0.58, 0.69	0.87	0.03	− 0.00, 0.07	0.06	0.08	− 0.42, 0.59	0.40
SFA*AG	0.04	0.18, 1.98	0.15	0.23	− 0.58, 1.04	0.57	− 0.02	− 0.07, 0.02	0.30	0.17	− 0.75, 0.39	0.10
*Crude*												
TFA*AA	101.72	− 1207.75, − 1.55	0.83	193.98	− 183.48, 571.44	0.31	82.87	− 325.99, 517.64	0.69	589.33	− 480.02, 1658.70	0.28
TFA*AG	175.09	− 879.14, 1082.59	0.89	393.89	− 620.30, 1408.09	0.44	66.45	− 1275.74, 1339.71	0.92	274.63	− 2594.84, 3144.10	0.85
*Adjusted*												
TFA*AA	649.22	397.61, 1696.05	0.22	34.29	− 46.25, 382.84	0.08	80.62	− 128.56, 94.47	0.36	556.93	− 476.72, 1590.59	0.09
	3.59	− 14.31, 21.50	0.69	3.10	− 3.24, 9.44	0.33						
TFA*AG	119.07	− 217.26, 455.41	0.48	168.84	− 632.04, 701.72	0.01	190.48	− 357.07, 738.04	0.49	652.71	− 2573.60, 387,903	0.69
	0.04	9.57	− 10.22, 29.37	0.34								
*Crude*												
MUFA*AA	0.15	− 0.01, 0.32	0.07	0.05	0.12, 2.56	0.10	0.07	− 0.01, 0.16	0.11	0.21	0.40, 4.70	0.03
MUFA*AG	− 0.00	− 0.17,0.16	0.96	0.00	− 0.06, 0.06	0.97	− 0.01	− 0.1, 0.09	0.85	− 0.04	− 0.22, 0.13	0.62
*Adjusted*												
MUFA*AA	0.02	− 0.36, 0.34	0.48	0.03	− 0.02, 0.08	0.22	0.03	− 0.00, 0.07	0.10	0.06	− 0.35, 0.47	0.36
MUFA*AG	0.00	− 0.00, 0.36	0.86	− 0.02	− 0.08, 0.02	0.30	− 0.01	− 0.06, 0.02	0.58	− 0.06	− 0.49, 0.39	0.33
*Crude*												
PUFA*AA	0.11	− 0.12, 0.36	0.34	0.06	− 0.03, 0.15	0.19	0.07	− 0.04, 0.19	0.24	0.18	− 0.08, 0.46	0.17
PUFA*AG	0.03	− 0.26, 0.19	0.04	0.00	− 0.08, 0.08	0.98	0.00	− 0.13, 0.13	0.98	− 0.07	0.17, 0.34	0.55
*Adjusted*												
PUFA*AA	0.06	− 0.39, 0.17	0.21	0.06	− 0.00, 0.13	0.06	0.04	− 0.01, 0.09	0.60	0.00	− 1.17, 0.17	0.62
PUFA*AG	0.01	− 0.10, 0.12	0.85	− 0.02	− 0.10, 0.04	0.46	− 0.01	− 0.07, 0.05	0.15	− 0.07	0.11, 0.59	0.98
*Crude*												
EPA and DHA*AA	6.41	− 12.2, 25.02	0.50	3.19	− 3.83, 10.22	0.37	1.28	− 10.02, 12.59	0.82	9.16	− 11.45, 29.79	0.38
EPA and DHA*AG	6.20	− 14.26, 26.68	0.55	3.45	− 4.26, 11.17	0.38	7.75	− 3.29, 18.80	0.16	5.32	− 17.35, 28.01	0.64
*Adjusted*												
EPA and DHA*AA	3.7	− 1.5, 9.02	0.16	1.85	− 9.63, 11.84	0.27	1.60	− 1.78, 2.13	0.25	4.89	− 3.61, 13.39	0.25
EPA and DHA*AG	2.37	− 3.71, 8.46	0.44	3.00	− 2.57, 18.49	0.12	1.59	− 1.43, 2.47	0.32	1.4	− 8.37, 11.26	0.77
*Crude*												
linoleic acid*AA	− 0.11	− 0.44, 0.21	0.49	0.01	− 0.11, 0.14	0.81	0.06	− 0.05, 0.19	0.28	0.10	− 0.27, 0.48	0.58
linoleic acid*AG	− 0.29	− 0.57, − 0.00	0.04	− 0.09	− 2.02, 0.01	0.10	0.01	− 0.11, 0.15	0.82	− 0.31	− 0.63, 0.01	0.05
*Adjusted*												
linoleic acid*AA	0.05	− 0.05, 0.16	0.31	0.06	− 0.01, 0.13	0.10	0.03	− 0.03, 0.10	0.27	− 0.01	0.16, 0.03	0.91
linoleic acid*AG	0.02	− 1.00, − 0.14	0.73	− 0.02	− 0.10, 0.05	0.52	0.00	− 0.07, 0.07	0.81	− 0.06	− 0.26, 0.12	0.48
*Crude*												
ALA*AA	− 2.97	− 8.43, 2.49	0.28	− 0.99	− 3.12, 1.14	0.36	− 0.01	− 2.85, 2.83	0.99	− 0.87	− 7.01, 5.26	0.78
ALA*AG	− 3.34	− 7.31, 0.64	0.10	− 1.21	2.75, 0.32	0.12	− 1.39	− 3.65, 0.86	0.22	− 1.23	− 3.19, 5.66	0.58
*Adjusted*												
ALA*AA	− 1.23	− 8.50, 6.04	0.74	− 0.81	− 3.91, 2.27	0.60	0.68	− 0.44, 1.81	0.23	− 0.55	− 6.17, 5.06	0.84
ALA*AG	− 3.96	− 10.49, 2.55	0.10	− 2.43	− 5.15, 0.29	0.12	− 0.38	− 1.40, 0.63	0.22	− 1.74	− 6.79, 3.29	0.58

	HC (cm)		*p* value	WHR		*p* value	WHtR		*p* value
	B	CI		B	CI		B	CI	
*Crude*									
Total fat*AA	0.02	− 0.08, 0.04	0.69	0.00	− 0.05, 0.06	0.99	0.00	0.00, 0.001	0.81
Total fat*AG	0.01	− 0.08, 0.13	0.78	− 0.004	− 0.11, − 0.00	0.90	0.00	− 0.00, 0.00	0.92
*Adjusted*									
Total fat*AA	3.66	0.03, 4.50	0.98	0.00	− 0.00, 0.00	0.46	0.00	0.00, 0.01	0.89
Total fat*AG	0.01	− 0.01, 0.04	0.32	0.00	− 0.00, 0.00	0.02	0.00	0.00, 0.00	0.30
*Crude*									
SFA*AA	0.05	− 0.11, 0.22	0.17	0.00	− 0.09, 0.1	0.95	0.00	− 0.17, 0.17	0.98
SFA*AG	0.06	− 0.14, 0.28	0.52	− 0.08	− 0.20, 0.04	0.18	− 0.00	0.00, 3.00	0.06
*Adjusted*									
SFA*AA	0.07	− 0.25, 0.40	0.59	0.00	− 0.00, 0.00	0.83	0.00	− 0.00, 0.00	0.55
SFA*AG	0.02	− 0.40, 0.35	0.73	0.00	− 0.00, 0.00	0.34	0.00	− 0.00, 0.00	0.18
*Crude*									
TFA*AA	23.86	− 931.24, 978.96	0.96	7.46	− 990.14, 1005.06	0.98	3.16	− 3.35, 9.69	0.34
TFA*AG	625.73	− 1937.12, 3188.59	0.63	5.12	− 5622.95, 1664.13	0.28	8.63	− 8.87, 26.13	0.33
*Adjusted*									
TFA*AA	38.07	− 647.26, 723.30	0.91						
TFA*AG	324.55	− 1814.39, 2463.49	0.76	4.84	− 0.88, 10.57				
*Crude*									
MUFA*AA	0.07	− 0.37, 0.41	0.37	0.00	− 0.09, 0.09	0.96	0.00	− 0.00, 0.00	0.14
MUFA*AG	0.11	0.25, 1.13	0.17	− 0.08	− 0.17, − 0.01	0.06	0.00	− 0.00, 0.001	0.40
*Adjusted*									
MUFA*AA	0.02	− 0.24, 0.29	0.85	0.00	− 0.00, 0.00	0.64	0.00	0.00, 0.00	0.94
MUFA*AG	0.03	− 0.25, 0.32	0.80	0.00	− 0.00, 0.00	0.04	0.00	− 0.00, 0.00	0.29
*Crude*									
PUFA*AA	0.06	− 0.17, 0.07	0.60	− 0.00	− 0.13, 0.13	0.98	0.00	− 0.00, 0.00	0.01
PUFA*AG	0.16	− 0.21, 0.37	0.13	− 0.14	− 0.26, − 0.01	0.02	− 0.00	− 0.00, 0.00	0.11
*Adjusted*									
PUFA*AA	0.01	− 0.43,0.23	0.46	0.00	− 0.00, 0.00	0.66	0.00	− 0.00, 0.00	0.56
PUFA*AG	− 0.01	− 0.00, 0.00	0.65	− 0.00	− 0.00, 0.00	0.32	− 0.00	− 0.00, 0.00	0.83
*Crude*									
EPA and DHA*AA	2.42	− 17.18, 22.02	0.80	0.03	− 13.00, 13.07	0.99	0.06	− 0.05, 0.19	0.28
EPA and DHA*AG	13.26	− 6.05, 32.58	0.17	6.38	− 7.95, 20.72	0.38	0.04	− 0.09, 0.17	0.53
*Adjusted*									
EPA and DHA*AA	− 0.07	− 3.69, 3.53	0.96	0.04	− 0.03, 0.12	0.25	0.01	− 0.2, 0.04	0.50
EPA and DHA*AG	0.43	− 3.74, 4.60	0.83	0.01	− 0.07, 0.10	0.76	0.02	− 0.02, 0.06	0.33
*Crude*									
linoleic acid*AA	− 0.10	− 0.43, 0.23	0.55	0.00	− 0.23, 0.23	0.99	0.00	− 0.00, 0.00	0.64
linoleic acid*AG	− 0.11	− 0.48, 0.25	0.55	− 0.21	− 0.42, − 0.01	0.03	− 0.00	− 0.00, 0.00	0.01
*Adjusted*									
linoleic acid*AA	0.01	− 0.09, 0.07	0.78	0.00	− 0.00, 0.00	0.81	0.00	− 0.00, 0.00	0.83
linoleic acid*AG	− 0.12	− 0.07, 0.07	0.78	− 0.00	− 0.00, 0.00	0.04	0.00	− 0.00, 0.00	0.37
*Crude*									
ALA*AA	− 0.87	− 7.01, 5.26	0.78	− 0.01	− 3.89, 3.93	0.99	0.00	− 0.03, 0.03	0.99
ALA*AG	− 1.23	− 3.19, 5.66	0.58	− 2.50	− 5.35, 0.34	0.06	− 0.03	− 0.05, − 0.00	0.03
*Adjusted*									
ALA*AA	− 0.55	− 6.17, 5.06	0.84	− 0.00	− 0.05, 0.04	0.76	− 0.02	− 0.07, 0.02	0.39
ALA*AG	− 1.74	− 6.79, 3.29	0.58	− 0.04	− 0.08, 0.00	0.06	− 0.04	− 0.09, − 0.00	0.07

All data are presented as B ± CI obtained from Generalized Linear Models

*p* value *: Adjusted for age, BMI, energy intake, and physical activity

*p* value < 0.05 was considered significant

*FM* fat mass, *WC* waist circumference, *HC* hip circumference, *WHR* weight to hip ratio, *WHtR* weight to height ratio, *SFA* saturated fatty acid, *TFA* trans-fatty acid, *MUFA* monounsaturated fatty acid, *PUFA* polyunsaturated fatty acid, EPA-DHA: eicosapentaenoic acid and docosahexaenoic acid, *ALA* alpha-linolenic acid

**Fig. 1:**
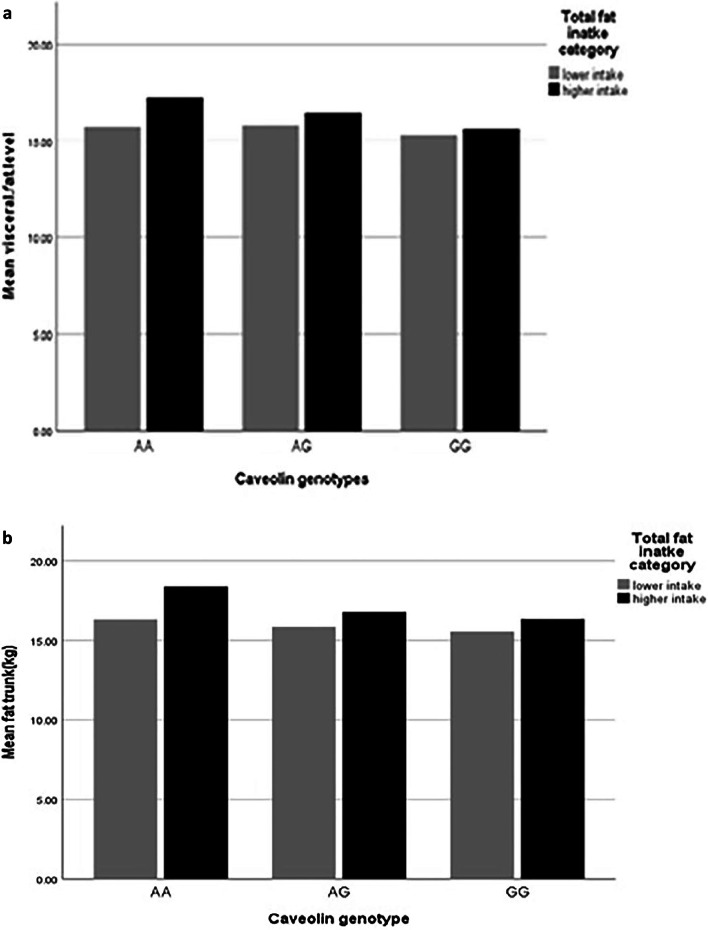

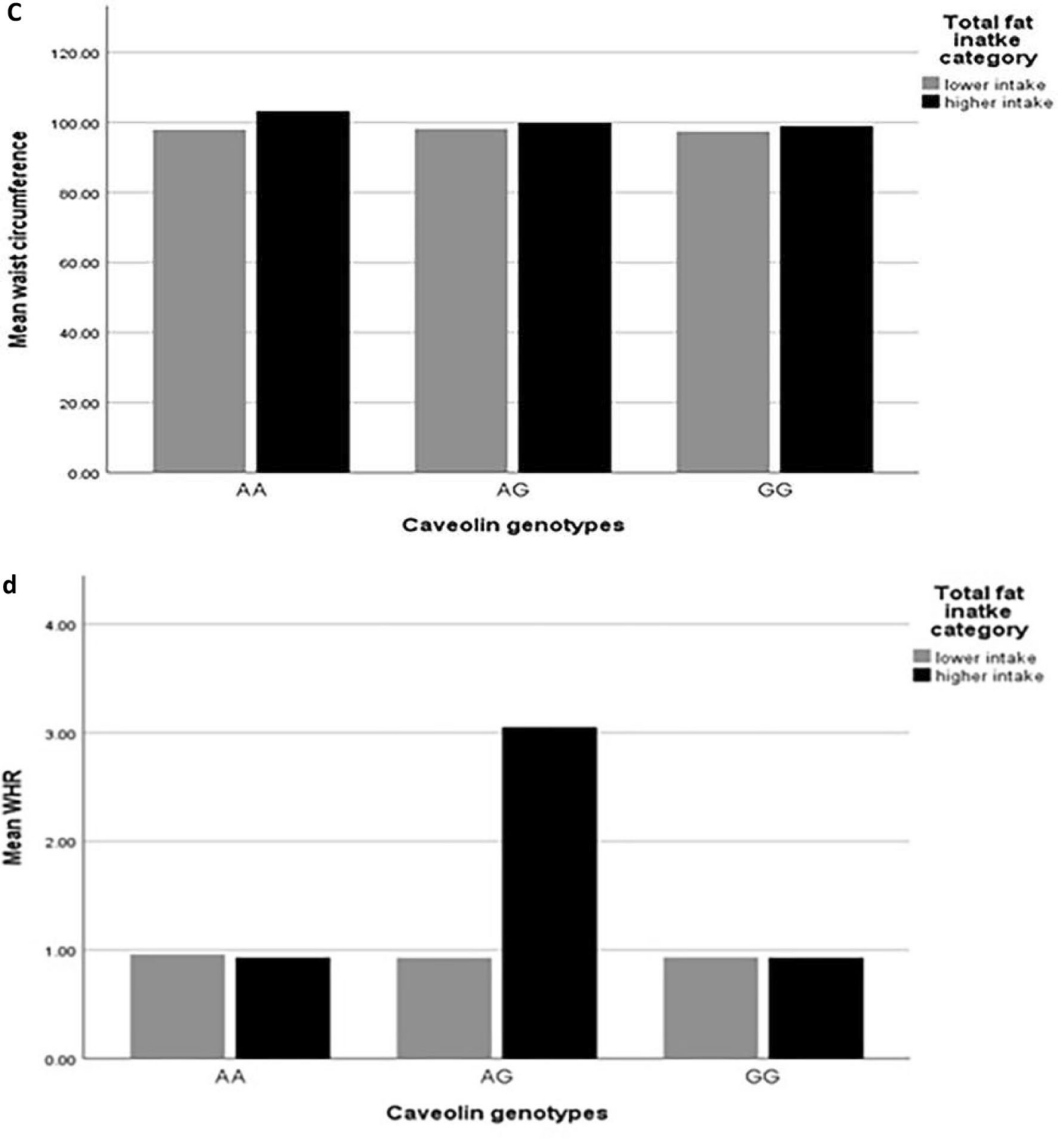

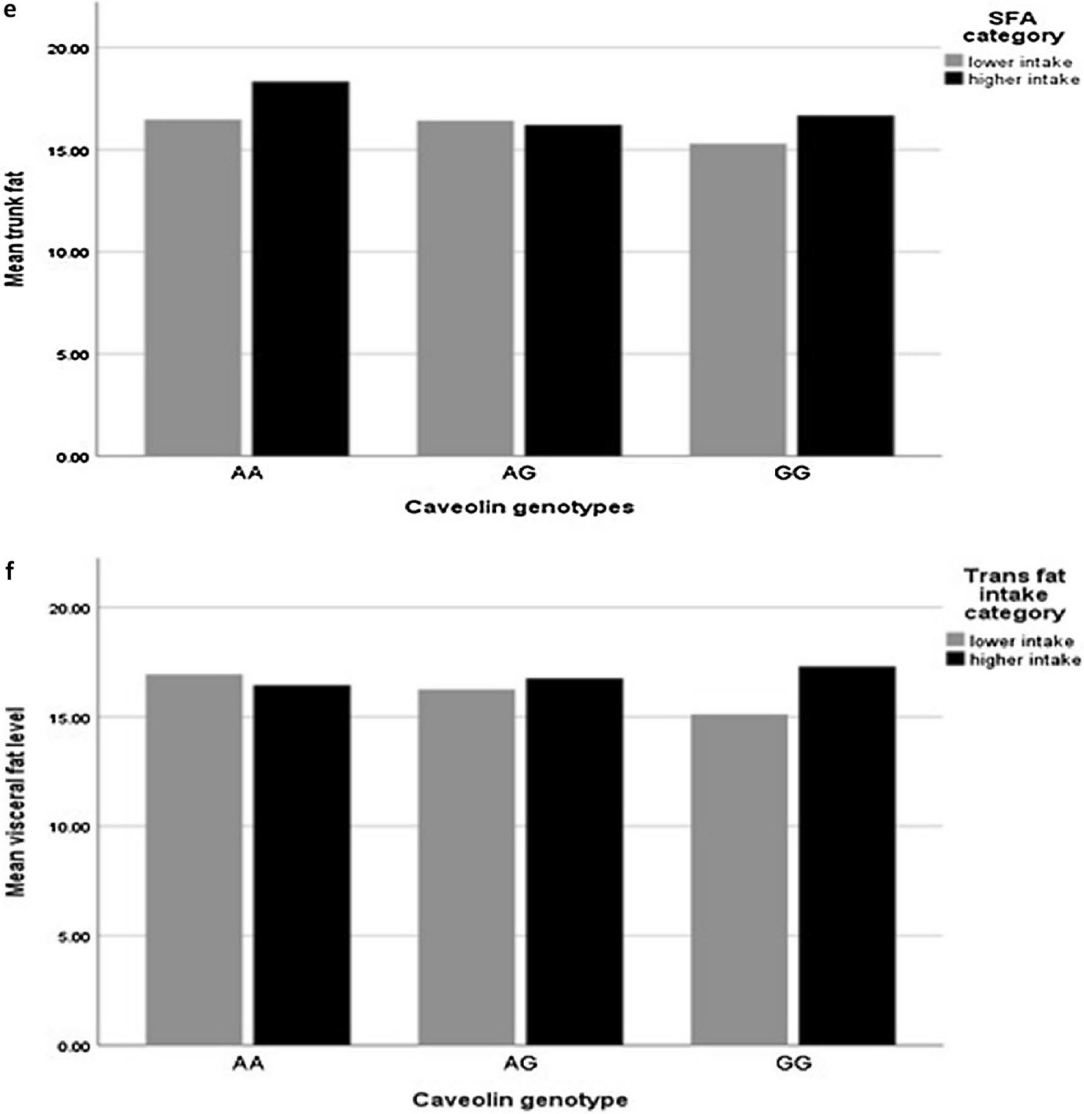

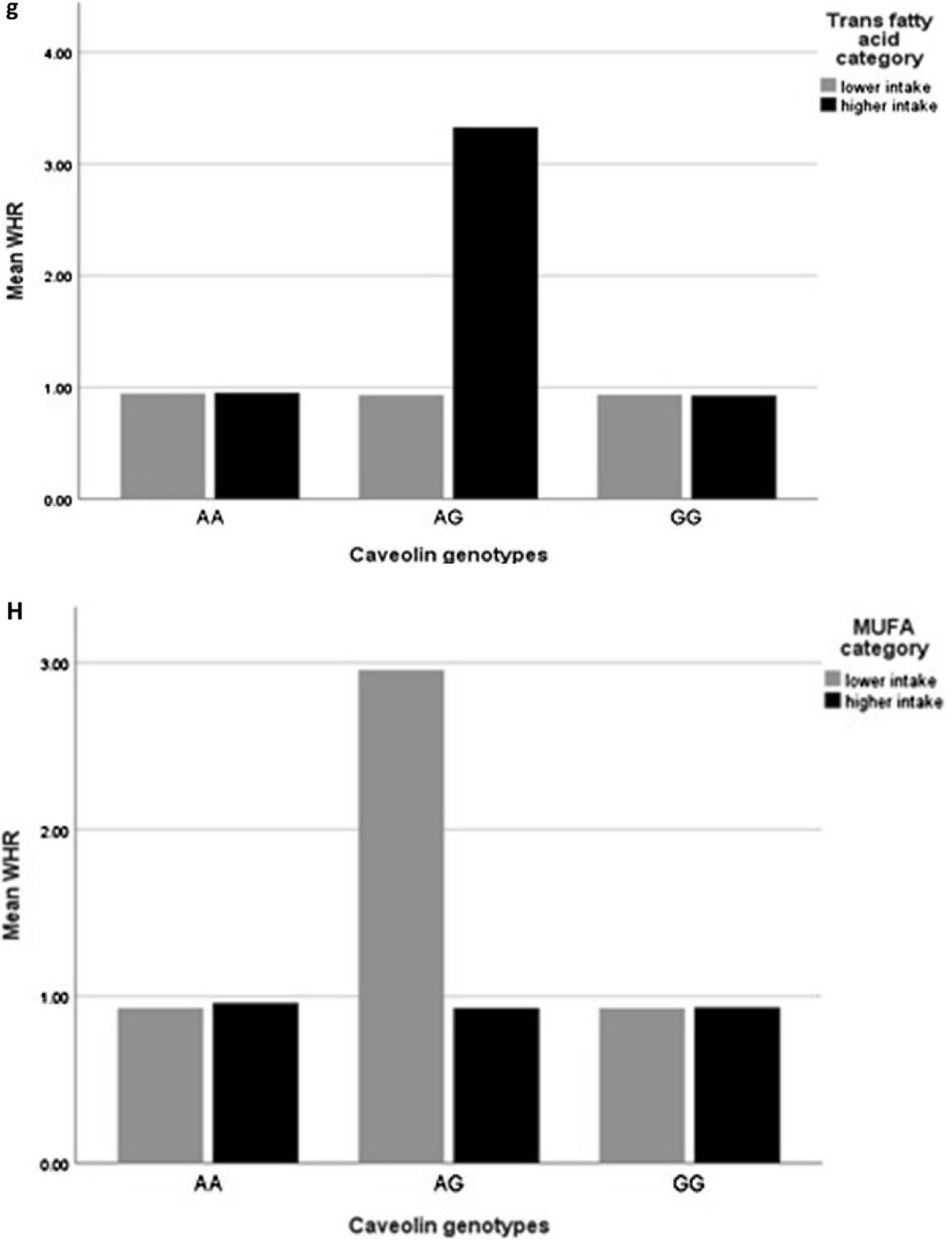

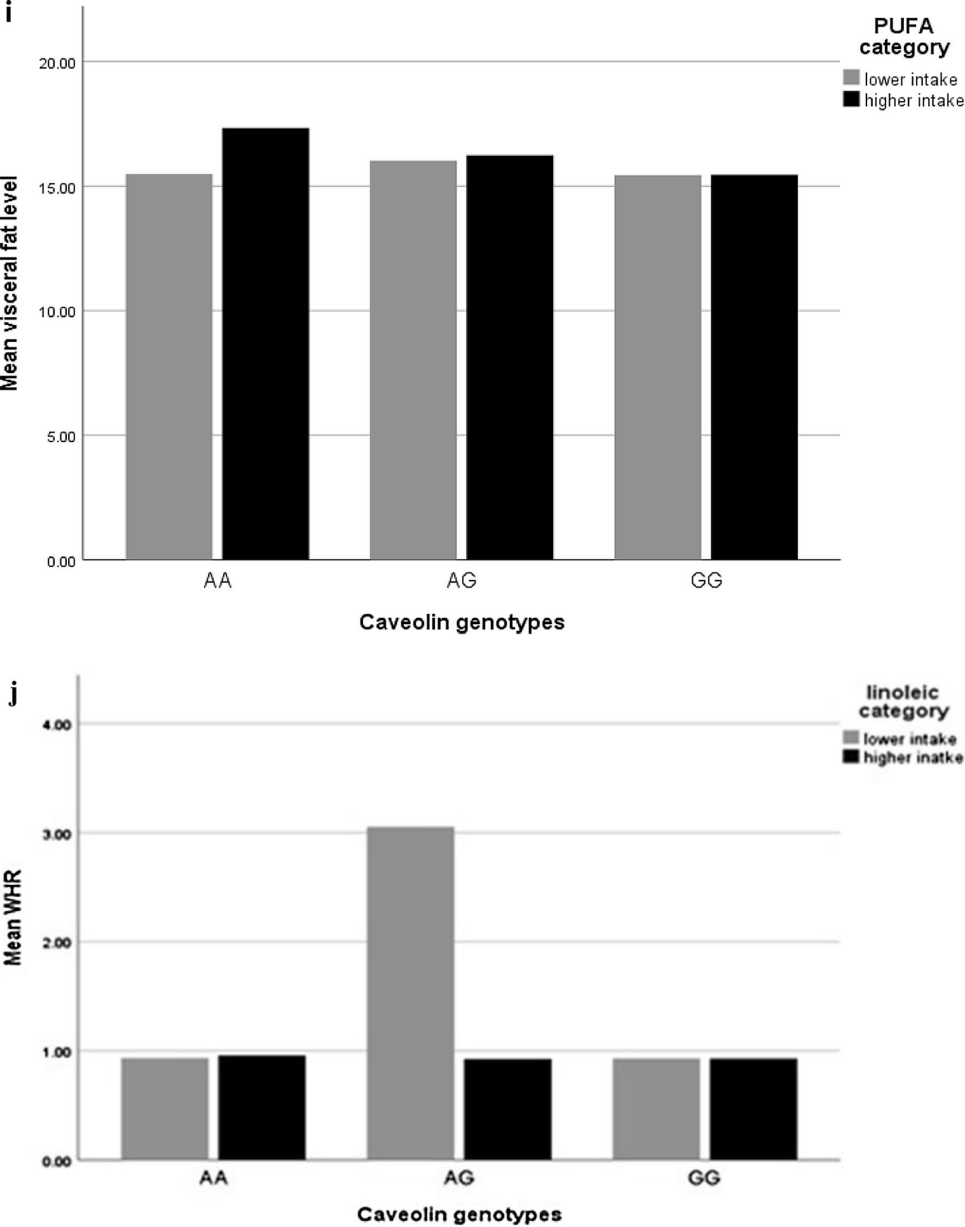

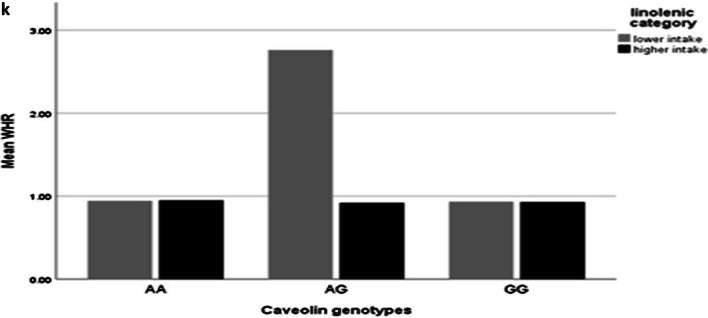
Interaction between rs 3807992 genotypes and dietary fats on the mean some of fat distribution variables. **a**–**d** With increasing total fat intake with two risk alleles (A), 0.11 cm^2^ vaisceral fat level (p-interaction: 0.86), 0.02 kg trunk fat (p-interaction: 0.76), 0.09 cm WC (p-interaction: 0.19) increased and that with increasing total fat intake with one risk alleles (A), 0.17 cm^2^ viseral fat level (p-interaction: 0.05) increased and 0.004 cm WHR decreased (p-interaction: 0.90). **e** With increasing SFA intake with having two risk alleles (A), 0.05 kg trunk fat increased (p-interaction: 0.23). **f**, **g** With increasing TFA intake with having one risk alleles (A), 5.12 cm WHR (p-interaction: 0.04) increased and 393.89 cm^2^ visceral fat level (p-interaction: 0.44) increased. **h** With increasing MUFA intake with having two risk alleles (A), 0.08 cm WHR (p-interaction: 0.28) decreased. **i** With increasing PUFA intake with having two risk alleles (AA), 0.06 cm^2^ visceral fat level (p-interaction: 0.19) increased. **j** With increasing linoleic acid intake with having one risk alleles (A), − 0.21 cm WHR (p-interaction: 0.03) decreased. **k** With increasing ALA intake with having one risk alleles (A), − 2.50 cm WHR (p-interaction: 0.06) decreased

After adjusting confounder variables (age, BMI, physical activity and energy intake), there was positive significant interaction between total fat intake and AA genotype on visceral fat level (β: 14.78; CI 5.71, 23.78; *p* value: 0.001) and trunk fat (β: 8.53; CI 6.20, − 3.61; *p* value: 0.01), and there was positive borderline significant interaction between total fat intake and AA genotype on WC (β: 0.00; CI − 0.06, 0.08; *p* value: 0.05), also there were positive significant interaction between total fat intake and AG genotype on WHR (β: 0.00; CI − 0.00, 0.00; *p* value: 0.02) and positive borderline significant interaction on visceral fat level (β: 7.53; CI − 7.20, 21.90; *p* value: 0.05) (Table 5, Fig. 1a–d).

### Interaction between SFA and *CAV-1* genotypes on fat distribution

In the crude model, there was positive significant interaction between SFA and AA genotype in comparison with the reference group (GG) on WC (β: 0.17; CI − 0.02, 0.36; *p* value: 0.04), and there was negative borderline interaction between SFA and AG genotype with WHtR (β: − 0.00; CI 0.00, 3.00; *p* value: 0.06).

After adjusting confounder variables (age, BMI, physical activity and energy intake), there was positive borderline significant interaction between SFA and AA genotype the trunk fat (β: 0.03; CI − 0.00, 0.07; *p* value: 0.06) (Table 5, Fig. 1e).

### Interaction between TFA and *CAV-1* genotypes on fat distribution

There was no significant interaction between rs 3807992 genotypes and trans-fatty acids on the body fat distribution, in the crude model (*p* value > 0.05).

After adjusting confounder variables (age, BMI, physical activity and energy intake), there was positive significant interaction between TFA and AG genotype in comparison with the reference group (GG) on WHR (β: 4.84; CI − 0.88, 10.57; *p* value: 0.04), and visceral fat level (β: 168.84; CI − 632.04, 701.72; *p* value: 0.01). There was no significant interaction between *rs 3807992* genotypes and TFA on other variables (*p* value > 0.05) (Table 5, Fig. 1f, g).

### Interaction between MUFA and *CAV-1* genotypes on fat distribution

In the crude model, there was a negative borderline interaction between MUFA and AG genotype in comparison with the reference group (GG) on WHR (β: − 0.08; CI − 0.17, − 0.01; *p* value: 0.06). There was no significant interaction between *rs 3807992* genotypes and MUFA on other variables (*p* value > 0.05).

After adjusting confounder variables (age, BMI, physical activity and energy intake), there was positive significant interaction between MUFA and AG genotype on WHR (β: 0.00; CI − 0.00, 0.00; *p* value: 0.04). There was no significant interaction between rs 3807992 genotypes and MUFA on other variables(*p* value > 0.05) (Table 5, Fig. 1h).

### Interaction between PUFA and *CAV-1* genotypes on fat distribution

In the crude model, there was negative significant interaction between PUFA and AG genotype in comparison with the reference group (GG) on WHR (β: − 0.14; CI − 0.26, 0.01; *p* value: 0.02) and positive significant interaction between PUFA and AA genotype on WHtR (β: 0.00; CI − 0.00, 0.00; *p* value: 0.01) and between PUFA and AG genotype on FM (β: 0.03; CI − 0.26, 0.19; *p* value: 0.04).

After adjusting confounder variables (age, BMI, physical activity and energy intake), there was a positive borderline interaction between PUFA and AA genotypes on visceral fat level (β: 0.06; CI − 0.00, 0.13; *p* value: 0.06) (Table 5, Fig. 1i).

### Interaction between EPA-DHA and *CAV-1* genotypes on fat distribution

Before and after adjusting confounder variables (age, BMI, physical activity, energy intake), there was no significant interaction between EPA and DHA and rs 3807992 genotypes on the body fat distribution indicators (*p* value > 0.05) (Table 5).

### Interaction between linoleic acid and *CAV-1* genotypes on fat distribution

In the crude model, there were negative significant interaction between linoleic acid and AG genotype in compare with the reference group (GG) on FM (β: − 0.29; CI − 0.57, − 0.00; *p* value: 0.04), WHR (β: − 0.21; CI − 0.42, − 0.01; *p* value: 0.03) and WHtR (β: − 0.002; CI − 0.00, 0.00; *p* value: 0.01) and borderline interaction between linoleic acid and AG genotype on WC (β: − 0.31; CI − 0.63, 0.01; *p* value: 0.05).

After adjusting for age, energy intake, physical activity and BMI, there was negative significant interaction between AG genotypes and linoleic acid on WHR (β: − 0.00; CI − 0.00, 0.00; *p* value: 0.04) (Table 4, Fig. 1j).

### Interaction between ALA and *CAV-1* genotypes on fat distribution

In the crude model, there was negative borderline significant interaction between ALA and AG genotype in comparison with the reference group (GG) on WHR (β: − 2.50; CI − 5.35, 0.34; *p* value: 0.06) and negative significant interaction on WHtR (β: − 0.03; CI − 0.05, − 0.00; *p* value: 0.03).

After adjusting confounder variables(age, BMI, physical activity and energy intake), there was negative borderline significant interaction between ALA and AG genotype on WHR (β: − 0.04; CI − 0.08, 0.00; *p* value: 0.06), so that with increasing ALA intake with having one risk alleles(A), 0.04 cm WHR decreased (Table 5, Fig. 1k).

## Discussion

This study investigated for the first time the simultaneous interaction of SNP rs 3807992 of the *CAV-1* genotypes and types of dietary fats intake in Iranian obese and overweight women.

There were an interaction between SFA and AA genotype with trunk fat, and between total fat intake and *CAV-1* genotype with visceral fat level, and between total fat intake and AA genotype with trunk fat and WC, and between total fat, MUFA, linoleic acid and ALA with AG genotype on WHR, and between SFA and AA genotype with trunk fat, and between PUFA and AA genotype with visceral fat level, and also between TFA and AG genotype with WHR and visceral fat level.

Studies have shown the quality and quantity of fat were related to changes in weight [43]. The results of our study did not show a relationship between dietary fat and body weight and were consistent with Field and Melanson studies, so that only a weak relationship was seen between total fat intake and body weight, and also there is not sufficient evidence regarding the impact of MUFA on the body weight [44, 45]. Other studies have found the opposite relationship between body weight with total dietary fat and its subtypes so that prospective cohort studies have shown a positive relation between TFA intake with weight changes, and also in a study that examined the relationship between SFA and MUFA on the body composition, have shown that significantly higher weight after the SFA than the MUFA-rich diet [45, 46]. A meta-analysis about the effect of reducing total fat intake on weight reported that lower energy intake in the low-fat group than in the control or usual fat groups suggested that a greater degree of energy reduction in the low-fat group was related to greater weight loss and weight reduction may be due to reduced energy intake in those on low-fat diets, rather than a specific effect of the macronutrient composition of the diet [47].

Our finding showed no relation between dietary fat and its subtypes with BMI and it is in agreement with Hu et al. so that there was no difference in BMI across tertiles of MUFA intake at baseline in the study [48]. Other studies have found the opposite relationship between BMI with total dietary fat and its subtypes so that the changes in percent dietary energy in the form of fat were positively related to changes in BMI [43]. There was a modest relation between a higher level of percentage of calories from fat and the long-term increase in BMI between overweight women with at least one overweight parent [45].

Also, our results in this study revealed no relationship between dietary fat and its subtypes with HC and this is not in consistent with Lofley's study so that there was a significantly relation between decrease change in HC and total carbohydrates, total fat, and vegetable fat intake [49].

This study indicated no relation between dietary fat and its subtypes with WC and it is in consistent with previous studies, including the Halkjær and Riserus study, so that animal fat and conjugated linoleic acid(CLA) had no effect on WC and the effect of CLA on WC not significantly different than the control group [50, 51]. This result is not in line with most previous studies and there was a relation between the increase in fat intake and vegetable fat and TFA with WC gain or an inverse relation between PUFA with WC, through changes in rate of oxidation and thermogenesis [51–53]. Hannon et al. showed that there was significant relation between WC and decrease in the SFA situation but yet conclusions cannot be made from these findings [54].

Our results indicated no relation between dietary fat and its subtypes with FM and it is in no agreement with previous studies so that in Kahleova et al. study showed there was a relation between reduced intake of SFA, TFA, or total fat with reduced FM [43]. A meta-analysis study has shown which there is significant relationship between high MUFA diets and decreases FM [55]. Results of studies have shown which there was a relation between changes in percent energy in the form of fat with percent of body fat remained significant even after adjustment for changes in BMI and changes in energy intake [43, 55].

Our study revealed no relation between dietary fats with visceral fat level and it is in consistent with Summers et al. study, so that in this clinical trial study in which individuals followed SFA and PUFA diets, total abdominal and visceral fat area were not affected by dietary change [56]. This result is not in agreement with most previous studies so that an intervention study in non-human primates indicates that high intake of TFA and without increasing total caloric intake caused visceral fat deposition and accumulation of fat in body, through increasing weight [57]. A systematic review study has shown abdominal fat decrease following consumption of high amounts of oleic acid-containing meal [58].

Our results found no correlation between dietary fat and its subtypes with trunk fat and leg or arm fat that it is in no agreement with Piers et al. study, so this study examined the relationship between SFA and MUFA on the body composition, the results showed trunk fat mass and limb fat mass were significantly greater after the SFA-rich diet [46].

That seems types of fat have different mechanical effects so that SFA intake, particularly compared to MUFA can reduce total lipid oxidation and energy expenditure [46, 59].

Our study showed that there was significant association between PUFA, linoleic acid, and ALA with CHOL/HDL and also significant association between SFA and insulin resistance that it is in agreement with most studies, including Park, Danielle, and Mozaffarian study so that showed there was a positive relation between PUFA and HDL-C and increased dietary omega 3 PUFA showed decreasing CHOL/HDL and also each 1% energy replacement of TFA with PUFA decreased CHOL/HDL by 0.67 [60–62]. A meta-analysis study showed that increasing the intake of PUFA instead of SFAs, in the long run, improves insulin resistance and MUFA intake, compared with SFA improves insulin sensitivity [63, 64]. Unsaturated fats change serum CHOL levels by mechanisms so that PUFA directly changes protein expression by upregulating mRNA levels and the number of cellular LDL receptors gains also reducing de novo lipogenesis and very-low-density lipoprotein secretion through fatty acid synthase suppression [65, 66].

There have been no studies on the relation of the *rs 3807992 CAV-1* gene on fat distribution, so we discuss related studies on the Cavolin gene and its polymorphisms. This study indicated a relation between *rs 3807992 CAV-1* gene with weight, BMI and HC that it is in agreement with Catalán et al. study so that there was a positive relation between *CAV-1* expression levels in Visceral adipose tissue and subcutaneous adipose with BMI [22]. Also, the results of the Abaj et al. study showed that participants with minor allele carriers had higher BMI, FMI and visceral fat levels [67]. This result is not in agreement with Mora-García et al. study so that there was no association between rs 926,198 and BMI [31].

Our results in this study showed an interaction between total fat intake and AA genotype with WC and also between total fat and AG genotype with WHR. There was an interaction between MUFA and AG genotype with WHR. There was an interaction between linoleic acid and ALA and AG genotype with WHR. No study has been performed on the interaction of *rs 3807992 CAV-1* and dietary fats with fat distribution. The results of the Yang et al. study showed that the high-fat diet was involved in the regulation of *CAV-1* [68]. The results of a study by Abaj et al. Showed that the A allele carriers were more odds of metabolic syndrome and its components (including abdominal obesity or high blood pressure) in individuals, and also, there was significant interaction between CAV 1 rs3807992 and SFA or PUFA on metabolic syndrome and its components [69]. The results of the Chung et al. study showed that the mean body in group fed a normal diet was lower than in group fed a high-fat diet, albeit in rats [70]. The study Mora-García et al. showed shown there was significant association between rs 926,198 and WHR but there was no association between rs 926198 and WC and WHtR [31]. The recessive allele of the rs3807998 variant increases the expression of the *CAV-1* gene, and increased expression of the *CAV-1* gene reduces the proliferation of endothelial cells and reduces the production of nitric oxide, and thus can increase the risk of metabolic syndrome disorders, and the expression of this recessive allele is higher in fat cells of obese people [71]. A cross sectional study showed that in people with recessive alleles, high intake of SFA or TFA increased the expression of the *CAV-1* gene [72].

Genetic differences, sample size, different confounding variables and even study design are some of the factors that can make the results of this study different from some articles.

Findings and results of this study can indicate opportunities for further studies in the future. Prospective and interventional studies in different populations and ethnicities should be performed to elucidate the effects of *CAV-1* and dietary fats onfat distribution and body composition. These findings need to be expanded to substantiate the results of this study clinically.

There are several strengths to this study. First, for the first time, it examines the interaction between dietary fats and genes and the pattern of body fat distribution. Second, used trained individuals to collect data and reduce bias. Third, the relationship between the subtypes of fat and body fat distribution was investigated. It also has several limitations, First of all, fill the FFQ questionnaire depends on the memory. Second, we could not evaluate the causal relation between dietary fats and *rs 3807992 CAV-1* with fat distribution due to the cross-sectional design. Third, this study was performed only on women therefore no generalisability. Fourth, medium sample size, and due to the low sample size and various confounding variables that could not be controlled by inclusion criteria, the confounding variables were adjusted in the statistical analyzes and some of these significant association were lost. But in fact, we will have the residual confounding effect on the results.

## Conclusion

The present study showed for the first time that the *CAV-1 rs 3807992* polymorphism and dietary fats is an important factor in improving the body composition of obese and overweight women. large studies in different populations should be conducted to elucidate the effect of CAV-1 and dietary fat on fat distribution pattern.

## Supplementary Information

**Additional file 1:** Image of gel electrophoresis.

## Data Availability

The all authors declare that the data supporting the results of this study are provided in this article, and all the data in the present study will be available with the opinion of the corresponding author for this study.

## Abbreviations

GWAS: Genome-wide association studies
*CAV-1*: Caveolin 1
SNP: Single nucleotide polymorphism
SFA: Saturated fatty acid
TFA: Trans-fatty acid
PUFA: Polyunsaturated fatty acid
MUFA: Monounsaturated fatty acid
ALA: Alpha-linolenic acid
EPA-DHA: Eicosapentaenoic acid and docosahexaenoic acid
CLA: Conjugated linoleic acid
WC: Waist circumference
HC: Hip circumference
NC: Neck circumference
FM: Fat mass
BMI: Body mass index
FMI: Fat mass index
WHR: Weight to hip ratio
WHtR: Weight to height ratio
HDL-C: High-density lipoprotein-cholesterol
LDL-C: Low-density lipoprotein-cholesterol
CHOL: Cholesterol
TG: Triglyceride
FBS: Fasting blood sugar
HOMA: Homeostasis model assessment
