# Supplementary material for: The interactions between dietary fats intake and Caveolin 1 rs 3807992 polymorphism with fat distribution in overweight and obese women: a cross-sectional study
Source: BMC Med Genomics. 2021 Nov 9;14:265. doi: 10.1186/s12920-021-01114-7 (PMC8579626; doi:10.1186/s12920-021-01114-7)
Supplement: Supplementary file 1 — Additional file 1: Image of gel electrophoresis. [file 12920_2021_1114_MOESM1_ESM.docx]

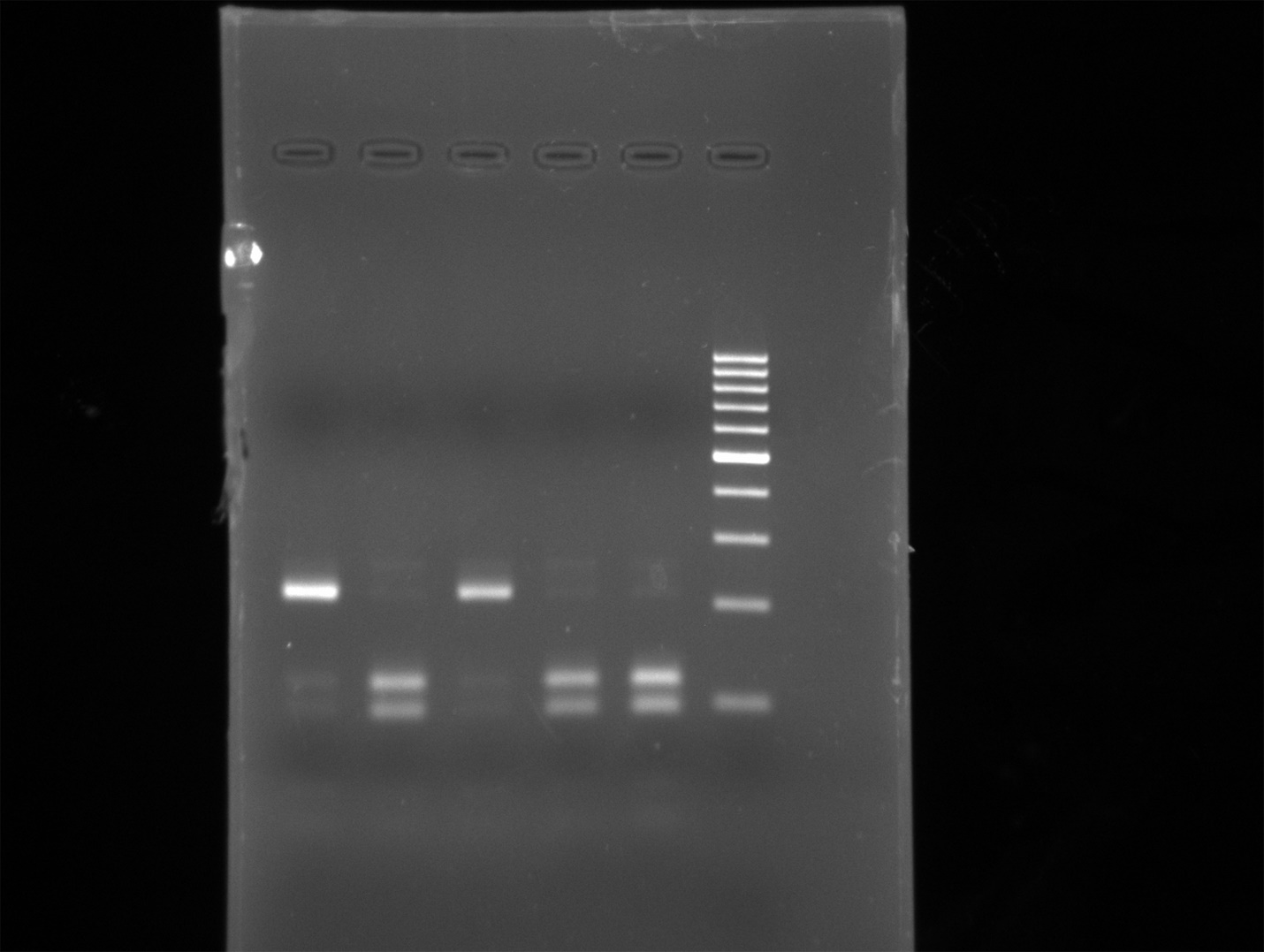


**Image of gel electrophoresis.** A Survey of rs3807992 CAV-1 polymorphism was conducted by PCR-RFLP and to ensure PCR performance, electrophoresis of PCR products was performed on the agarose gel. Pieces containing 3 genotypes were distinguished: GG, AA, and AG.
